# Inhibition of tryptophan 2,3-dioxygenase impairs DNA damage tolerance and repair in glioma cells

**DOI:** 10.1093/narcan/zcab014

**Published:** 2021-04-09

**Authors:** Megan R Reed, Leena Maddukuri, Amit Ketkar, Stephanie D Byrum, Maroof K Zafar, April C L Bostian, Alan J Tackett, Robert L Eoff

**Affiliations:** Department of Biochemistry and Molecular Biology, University of Arkansas for Medical Sciences, Little Rock, AR 72205, USA; Department of Biochemistry and Molecular Biology, University of Arkansas for Medical Sciences, Little Rock, AR 72205, USA; Department of Biochemistry and Molecular Biology, University of Arkansas for Medical Sciences, Little Rock, AR 72205, USA; Department of Biochemistry and Molecular Biology, University of Arkansas for Medical Sciences, Little Rock, AR 72205, USA; Arkansas Children's Research Institute, 1 Children’s Way, Little Rock, AR 72202, USA; Department of Biochemistry and Molecular Biology, University of Arkansas for Medical Sciences, Little Rock, AR 72205, USA; Department of Biochemistry and Molecular Biology, University of Arkansas for Medical Sciences, Little Rock, AR 72205, USA; Department of Biochemistry and Molecular Biology, University of Arkansas for Medical Sciences, Little Rock, AR 72205, USA; Arkansas Children's Research Institute, 1 Children’s Way, Little Rock, AR 72202, USA; Department of Biochemistry and Molecular Biology, University of Arkansas for Medical Sciences, Little Rock, AR 72205, USA

## Abstract

Expression of tryptophan 2,3-dioxygenase (TDO) is a determinant of malignancy in gliomas through kynurenine (KYN) signaling. We report that inhibition of TDO activity attenuated recovery from replication stress and increased the genotoxic effects of bis-chloroethylnitrosourea (BCNU). Activation of the Chk1 arm of the replication stress response (RSR) was reduced when TDO activity was blocked prior to BCNU treatment, whereas phosphorylation of serine 33 (pS33) on replication protein A (RPA) was enhanced—indicative of increased fork collapse. Analysis of quantitative proteomic results revealed that TDO inhibition reduced nuclear 53BP1 and sirtuin levels. We confirmed that cells lacking TDO activity exhibited elevated gamma-H2AX signal and defective recruitment of 53BP1 to chromatin following BCNU treatment, which corresponded with delayed repair of DNA breaks. Addition of exogenous KYN increased the rate of break repair. TDO inhibition diminished SIRT7 deacetylase recruitment to chromatin, which increased histone H3K18 acetylation—a key mark involved in preventing 53BP1 recruitment to sites of DNA damage. TDO inhibition also sensitized cells to ionizing radiation (IR)-induced damage, but this effect did not involve altered 53BP1 recruitment. These experiments support a model where TDO-mediated KYN signaling helps fuel a robust response to replication stress and DNA damage.

## Graphical Abstract

**Graphical abstract:**
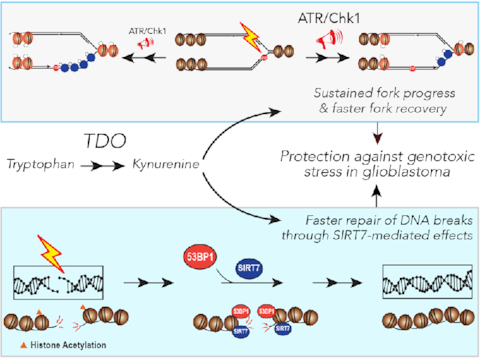
The replication stress and DNA damage responses in glioma cells are modulated by tryptophan 2,3-dioxygenase activity, a key factor controlling kynurenine signaling and *de novo* synthesis of NAD+.

## INTRODUCTION

Glioblastoma multiforme (GBM or simply glioblastoma) represents an especially deadly type of primary brain tumor afflicting both adult and pediatric patients (1–3). The usual course of treatment includes surgery followed by a combination of ionizing radiation (IR) and chemotherapy with DNA alkylators temozolomide (TMZ or Temodar) and bis-chloroethylnitrosourea (BCNU or Carmustine) being two of the most common adjuvant therapies (4). Yet, outcomes for patients with high-grade gliomas have not dramatically improved. The challenges to effective treatment of glioblastoma are many and include the location of the tumor, invasive microtubes, tumor heterogeneity, a relatively high proportion of tumor initiating (or stem-like) cells, and a robust replication stress/DNA damage response (RSR/DDR) capacity (5–8). The factors driving increased tolerance of DNA damage and replication stress (RS) are likewise multi-factorial and strongly correlated with resistance to genotoxic drugs and tumor recurrence in glioblastoma patients (9).

The aberrant and constitutive degradation of tryptophan to kynurenine (KYN) and subsequent activation of the aryl hydrocarbon receptor (AhR)—a ligand-activated transcription factor involved in a variety of biological processes—has emerged as a driving force in multiple aspects of GBM biology (10). One route to KYN pathway (or KP) activation in GBMs occurs in response to COX2/prostaglandin E_2_-mediated up-regulation of tryptophan 2,3-deoxygenase (TDO2 or TDO) (11). TDO is one of three human enzymes catalyzing the rate-limiting step in the conversion of tryptophan to KYN. In GBM and advanced stage breast cancer, TDO promotes a pro-malignant/anti-immune response through production of KYN, an endogenous agonist of the AhR transcription factor, and other tryptophan catabolites. The KP-AhR cascade produces different effects on tumor and immune cells. To date, the majority of studies on the role of the KP in cancer have focused on immunosuppressive effects. The use of epacadostat (an inhibitor of KP signaling) in combination with Merck's anti-PD-1 antibody pembrolizumab in phase III clinical trials for the treatment of advanced stage melanoma highlights the interest in targeting the KP as an adjuvant to immune checkpoint inhibitors (12), although it remains uncertain if targeting KP-related enzymes is a useful therapeutic strategy (13).

Previously, we showed that modulation of KP-AhR signaling affected the expression of the RSR enzyme DNA polymerase kappa (hpol κ) (14). Treating GBM-derived cell lines with an inhibitor of TDO lowered hpol κ expression and led to a diminished level of micronuclei (MN). The same was true for GBM cells treated with either the AhR antagonist CH-223191 or siRNA against hpol κ. Combined inhibition/knockdown of either TDO and AhR or TDO and hpol κ did not decrease MN levels further—suggestive of an epistatic relationship. Since hpol κ performs multiple functions related to DNA damage tolerance and the resolution of RS and because the AhR is a transcription factor known to regulate a variety of pathways, we postulated that attenuation of the KP might sensitize GBM cells to genotoxic drugs by changing the basal RSR/DDR capacity.

In the current study, we have investigated the hypothesis that RSR and DDR programs in glioma-derived cells are responsive to KP signaling specifically through TDO activity and that this connection modulates sensitivity to RS and DNA damage. We tested this idea by measuring TDO-dependent changes to DNA replication and repair in glioma-derived cells treated with either hydroxyurea (HU) or the DNA damaging agent BCNU. The relationship between TDO activity and response to IR was also addressed. We focused on analyzing changes related to RSR/DDR activation and fork dynamics, as well as more direct readouts for genomic integrity (e.g. alkaline comet assay and micronucleation assay). Cell cycle progression, viability and motility were also assessed. Quantitative proteomic analysis was used to evaluate TDO-related changes in an unbiased and global manner. Cumulatively, our *in vitro* results are consistent with the notion that activation of KYN signaling increased the threshold for tolerance of DNA damage and RS in GBM cells—a phenomenon that could have implications for genotoxic therapies.

## MATERIALS AND METHODS

### Chemicals

All chemicals were molecular biology grade or better. L-kynurenine (KYN; Cat# K8625) and Carmustine (BCNU; Cat# C0400) were purchased from Sigma-Aldrich (St. Louis, MO) and resuspended in water or 100% ethanol (EtOH), respectively. The small molecule inhibitor of TDO (680C91; Cat# 4392) was purchased from Tocris Bioscience (Bristol, UK) and resuspended in dimethyl sulfoxide (DMSO). For all treatment conditions, final concentration of DMSO/EtOH used was <1% (v/v). Experiments were performed with multiple biological replicates, where appropriate experimental treatments were randomized, and all immunofluorescence scoring was conducted in a blinded manner. Statistical evaluations are reported in the figure legends. A detailed list of key resources (e.g. antibodies and siRNA) can be found in the supporting information (Supplementary Table S1).

### Cell culture

The glioblastoma-derived cell line T98G was obtained from the American Type Culture Collection (ATCC; Cat# CRL-1690, Manassas, VA). The glioblastoma-derived cell line U-251MG was obtained from the European Collection of Authenticated Cell Cultures (ECACC) (Sigma-Aldrich; Cat# 09063001, St. Louis, MO). Cells were maintained (5% v/v CO_2_, 37°C) in Modified Eagle’s medium (MEM) containing 10% (v/v) fetal bovine serum and 1% (v/v) antibiotic/antimycotic containing 100 U/ml penicillin, 100 μg/ml streptomycin and 0.25 μg/ml amphotericin B (Sigma-Aldrich, St. Louis, MO).

### Cell viability assay

T98G cell viability was measured using the Calcein AM assay, where 5 × 10^3^ cells were plated per well in a 96-well dish. Cells were treated with 680C91 (10 and 20 μM) for 24 h. Cells were treated for with varying concentrations of BCNU (0–2 mM) for an additional 48 h before incubation with Calcein AM dye (2 μM) (Invitrogen; Cat# C1430, Grand Island, NY) at 25°C for 30 m. To determine percent viability, fluorescence values were obtained using Synergy4 plate reader at an excitation wavelength 485 nm and emission wavelength 528 nm. The EC_50_ values were calculated by fitting the resulting data to a four-parameter logistic model using Prism software.

### Clonogenic assay

A six-well dish was plated with 500 cells per well, and cells were allowed to adhere for 24 h. Depending on experimental design, wells were either immediately treated with 10 or 20 μM of 680C91, 60 μM KYN and 125 μM BCNU for 1 h or pretreated with 680C91/KYN 24 h prior to BCNU treatment. Cells were treated with BCNU (125 μM) for 1 h before replacing with fresh media and allowing cells to recover for 8–10 days. Cells were fixed with formaldehyde (3.7% v/v) before staining with crystal violet (Sigma Aldrich; Cat# V5265, St. Louis, MO). For ionizing radiation experiments, cells were plated in a 10 cm^2^ dish and either pretreated with media that contained 20 μM 680C91 or media with DMSO alone for 24 h prior to irradiation. Cells were then either mock-treated or exposed to 2, 5 or 10 Gy IR before being replated into six-well dishes at a density of 2000 cells per well. Cells were grown for an additional 12 days prior to fixation and staining with crystal violet. Colonies were counted using an EVOS FL Auto microscope (Life Technologies, Carlsbad, CA). A colony was scored if at least 25 cells were present. Colony diameter was also measured. For all clonogenic survival assays, the plating efficiency for the control reaction was determined and the surviving fraction was calculated by dividing the number of colonies in the treated condition by the product of multiplying the number of cells plated by the plating efficiency divided by 100. Statistical comparisons were made using a one-way ANOVA with a Tukey post-test.

### Flow cytometry

Cells were stained with Click-it EdU Imaging Kit (ThermoFisher Scientific; Cat# C10337, Waltham, MA) using the following protocol. Cells were treated with 680C91 (10 or 20 μM) or Kyn (60 μM) 24 h prior to BCNU treatment. Cells were treated with BCNU (125 μM) for 25 h prior to EdU labeling (10 μM) for 1 h, harvested, washed and fixed in 70% (v/v) EtOH before adding permeabilization buffer containing 0.5% (w/v) Triton X-100 in 1× PBS and incubating at RT for 30 min. Cells were washed and incubated in click-it reaction cocktail (click-it reaction buffer, CuSO_4_, 1x click-it reaction buffer additive, Alexa Fluor 647) for 30 min in the dark at RT. Propidium Iodide (PI) stain (BD Biosciences; Cat# 556463, San Jose, CA) was added for 1 h prior to analysis. During flow analysis, azide only control cells were used for gating. Representative scatterplots for each condition are shown in Supplementary Figure S1.

### Immunofluorescence

Cells were treated with 680C91 (20 μM) or KYN (60 μM) 24 h prior to BCNU treatment. Cells were treated with BCNU (125 μM) for 24 h before fixing with 3.5% (v/v) formaldehyde and permeabilized with 1× PBS containing 0.2% (w/v) Triton X-100, 0.01% (w/v) sodium azide, 100 μg chicken ovalbumin (Sigma Aldrich; Cat# A2512, St. Louis, MO) and stained for RPA2 (pS33) or γH2AX. Experiments were repeated with in biological triplicate, with at least 130 cells quantified per condition. For the pre-extracted experiments, cells were treated with 0.5% (w/v) Triton-X-100 in 1xPBS on ice for 5 min, prior to fixation with 3.5% (v/v) formaldehyde. Coverslips were permeabilized as described above and co-stained with 53BP1 and γH2AX (catalogue numbers and antibody dilutions can be found in Supplementary Table S1). Experiments were repeated in biological duplicate, with at least 159 cells quantified per condition. For EdU pulse experiments, cells were labeled with EdU (10 μM) alone or concurrently with BCNU (125 μM) or HU (2 mM) for 1 h. Cells were fixed and permeabilized as described above, before cells were stained with Alexa Fluor 488-azide (Thermo Fisher Scientific; Cat# A10266, Waltham, MA) via a click chemistry reaction. Click reaction buffer was comprised of 1× PBS, 25 μM 488-azide, 20 mg/ml sodium ascorbate and 20 μM CuSO_4._ Coverslips were incubated in click reaction buffer for 30 min at room temperature. Experiments were repeated in biological duplicate, with at least 300 cells quantified per condition.

For ionizing radiation (IR) experiments, cells were plated on coverslips and a Mark 1 model 68A cesium-137 source (J.L. Shepherd) was used to expose cells to γ-rays at a dose of 0.94 Gy/min, to a total dose of 2 or 10 Gy. Cells were pre-extracted with 0.5% (w/v) Triton-X-100 prior to fixation with 3.5% (v/v) formaldehyde at 2, 6 and 24 h post irradiation. Fixed cells were stained for γH2AX and 53BP1 using antibodies and dilutions found in Supplementary Table S1. At least 400 cells were scored per condition from two biological replicates. All experiments were imaged via Olympus Fluoview FV100 microscope and blind scored via ImageJ and Cell Profiler. Statistical analysis was performed using a one-way ANOVA with a Tukey post-test.

### Immunoblotting

T98G cells were treated with 680C91 or KYN alone, or in combination with BCNU exactly as described earlier, and subsequently processed for immunoblotting. Whole cell lysates (WCL) of T98G cells were prepared by resuspending the frozen cell pellets in 40 mM HEPES (pH 7.5) buffer containing 120 mM NaCl, 1 mM EDTA, 50 mM NaF and 1% (w/v) Triton X-100. A 1× dilution of protease inhibitor cocktail (Sigma-Aldrich; Cat# P8340, St. Louis, MO) was added to the lysis buffer, as well as the phosphatase inhibitors sodium β-glycerophosphate and sodium orthovanadate. Lysis was achieved by incubation on ice with intermittent mixing for 1 h. This was followed by pelleting the cell debris in a centrifuge by spinning at 21 130 × *g* for 15 min at 4°C. Prior to submission to the UAMS proteomics core facility, harvested cells were separated into nuclear and cytosolic fractions via NE-PER kit following the manufacturer’s instructions (ThermoFisher Scientific; Cat# 78833, Waltham, MA).

To analyze changes in the amount of protein bound to chromatin, we fractionated soluble nuclear and chromatin bound (CB) proteins. Cells were harvested with cell a cell scraper and resuspended in 10 mM HEPES (pH 7.9) buffer containing 10 mM KCl, 1.5 mM MgCl_2_, 0.34 M sucrose, 10% (v/v) glycerol, 0.1% (w/v) Triton X-100, 1 mM DTT, 1 mM NaF, 1 mM Na_3_VO_4_ and 1× protease inhibitor cocktail. Cells were incubated in lysis buffer for 5 min on ice before centrifugation at 1300 × *g* for 4 min. Nuclei were obtained in the pellet and further separated into soluble and chromatin bound fractions by incubating nuclei in buffer containing 3 mM EDTA, 0.2 mM EGTA, 1 mM DTT, 1 mM NaF, 1 mM Na_3_VO_4_ and 1× protease inhibitor for 10 min on ice. Supernatant containing soluble nuclear proteins was collected after centrifugation at 1700 × *g* for 4 min. The pellet containing chromatin bound proteins was resuspended in 5× SDS loading dye and boiled for 5 min at ∼90°C. To get the chromatin pellet into solution, boiled sample was subjected to water bath sonication until able to easily pipette (∼5 min of 30 s on/ 30 s off on maximum power). Total protein concentration of both the WCL and CE samples was estimated by a colorimetric bicinchonic acid (BCA) assay (Thermo Scientific; Cat# 23225, Waltham, MA), using a bovine serum albumin as a standard, as per manufacturer’s instructions.

Proteins from WCL samples were separated on 4–20% (w/v) Tris-Glycine SDS-PAGE gels (Bio-Rad; Cat#456–1094, Hercules, CA). We loaded 30–50 μg protein per sample, and the proteins were separated by electrophoresis under a constant voltage of 120 V for 60–90 m. The gel-separated protein bands were transferred on to a 0.2 μm nitrocellulose (Bio-Rad; Cat# 162–0112, Hercules, CA) or polyvinylidene difluoride (PVDF; Bio-Rad; Cat# 162–0177, Hercules, CA) membrane under constant current of 0.2 A for 60–75 min in an ice-bath. The CE samples were subjected to immunoblotting using the same protocol as described above, when probing for all proteins other than ATM and ATR. The gel-running and transfer protocols were modified for the blots probed for the high molecular weight proteins (>250 kDa) ATM and ATR in the CE samples as follows: the samples were loaded on 4–8% (w/v) Tris-acetate SDS-PAGE gels (ThermoFisher Scientific; Cat# EA03752BOX, Waltham, MA). The running buffer and transfer buffer were prepared as per manufacturer’s instructions. PVDF membrane was used for these blots, and transfer was performed at a constant current of 0.35 A for 4 h at 4°C. After transfer was completed, the blots were incubated in a blocking solution containing 5% (w/v) dried milk solution in 1× Tris-buffered saline (TBS) for 1 h. After blocking, the blots were incubated overnight with the suitable primary antibody on a shaker at 4°C (a complete list of all the primary and secondary antibodies used for immunoblotting is provided in the antibodies resources table). The following day, the blots were washed twice in 1× TBS containing 0.1% (v/v) Tween-20 (TBST), and then incubated in an HRP-conjugated secondary antibody on a shaker at room temperature. After washing twice with TBST, the blots were developed by enhanced chemiluminescence (ECL) using a kit (Bio-Rad; Cat# 1705060, Hercules, CA) and the protein bands were visualized on a ChemiDoc digital imager (Bio-Rad; Cat# 12003153, Hercules, CA). Detailed antibody information can be found in Supplementary Table S1. Uncropped immunoblots are shown in Supplementary Figures S2 and S3.

### Micronucleation

Micronucleation experiments were performed as previously described (14). Briefly, cells were treated with 680C91 (20 μM) or KYN (60 μM) 24 h prior to BCNU treatment. Cells were treated with BCNU (125 μM) for 24 h before treating with cytochalasin-B (2 μg/ml) (Sigma Aldrich; Cat# 250233, St. Louis, MO) for an additional 24 h. Slides were stained with α-tubulin. Detailed antibody information can be found in Supplementary Table S1. Images were obtained via Olympus Fluoview FV100 microscope and subjected to blinded scoring in biological triplicate.

### DNA fiber spreading

Cells were pretreated with 680C91 (20 μM) or KYN (60 μM) 24 h prior to start of experiment. Pretreated cells were incubated with media containing 20 μM 5-chloro-2′-deoxyuridine (CldU) (Milipore Sigma; Cat# C6891, St. Louis, MO) for 30 m at 37°C before being washed and incubated in media containing 100 μM 5-iodo-2′-deoxyuridine (IdU) (Milipore Sigma; Cat# I7125, St. Louis, MO) alone or in combination with BCNU (125 μM) for 1 h at 37°C. Fork restart experiments were also pretreated as described above followed by 3 mM hydroxyurea (HU) (Milipore Sigma; Cat# H8627, St. Louis, MO) treatment for 4 h after CldU treatment and then 30 min IdU pulse. Cells were counted and diluted to 1 million cells/ml and mixed 1:1 with unlabeled cells. Around 2 μl of cell suspension was dropped onto top of glass slides and solution was left to air dry until volume was slightly reduced before mixing in DNA lysis buffer (0.5% (w/v) SDS, 200 mM Tris-HCl pH 7.4, 50 mM EDTA) and incubating for 5 min at RT. Slides were then tilted to ∼15° for the solution to reach to bottom of the slides and then dried. Dried slides were fixed in 3:1 dilution of methanol and acetic acid for 3 min before incubating in 2.5 M HCl for 70 min. Slides were washed and transferred into a humid box where they were incubated in blocking buffer (10% (v/v) goat serum in 1× PBS) for 60 min at RT. Slides were stained for CldU and IdU for 2 h at RT before adding corresponding secondary antibodies for an additional h at RT. The length of IdU and CldU was measured for each fiber counted and normalized to analogue incubation time (60 or 30 min, respectively). Detailed antibody information can be found in Supplementary Table S1. Images were obtained via Nikon Ti2 Eclipse microscope and subjected to blinded scoring in biological triplicate. Statistical analysis performed was a non-parametric Mann–Whitney analysis of means.

### siRNA knockdown

Cells were transfected with On-TARGETplus pooled siRNA oligonucleotides at a final concentration of 10 nM (40 pmol siRNA) by combining with Lipofectamine 3000 transfection reagent (ThermoFisher Scientific; Cat#L3000008, Waltham, MA) and Opti-MEM reduced serum media (ThermoFisher Scientific; Cat# 31985062, Waltham, MA) per manufacturer’s instructions. Knockdown efficiency was examined via immunoblotting for TDO at 72 h post transfection. For comet assay, transfected cells were harvested at 48 h post transfection and plated at a confluency of 100 000 cells per well of a 6-well dish. Transfected cells were pretreated with 60 μM KYN (where applicable) for 24 h and then treated with BCNU (125 μM) for an additional 24 h. Detailed siRNA oligonucleotide information can be found in Supplementary Table S1.

### FASP bHPLC TMT Mass Spectrometry

T98G cells (∼10^6^ cells/condition) were pretreated with 680C91 (10 or 20 μM) or cultured in DMSO containing media as a control for 24 h prior to BCNU treatment. After the 680C91 pre-treatment, the media were replaced with fresh media containing DMSO, 680C91 alone or BCNU (125 μM). After 24 h treatment with BCNU, cells were harvested and subjected to nuclear and cytosolic fractionation via NE-PER Kit (ThermoFisher Scientific; Cat# 78833, Waltham, MA). Nuclear protein was isolated and 100 μg of lysate was submitted to UAMS proteomics core facility.

Purified proteins were reduced, alkylated and digested using filter-aided sample preparation (15). Tryptic peptides were labeled using a tandem mass tag 10-plex isobaric label reagent set (ThermoFisher Scientific, Cat# 90110, Waltham, MA) following the manufacturer’s instructions. Labeled peptides were separated into 36 fractions on a 100 × 1.0 mm Acquity BEH C18 column (Waters, Cat# 186002346, Milford, MA) using an UltiMate 3000 UHPLC system (ThermoFisher Scientific, Waltham, MA) with a 40 min gradient from 99:1 to 60:40 buffer A:B ratio under basic pH conditions, and then consolidated into 12 super-fractions. Each super-fraction was then further separated by reverse phase XSelect CSH C18 2.5 um resin (Waters, Cat# 186006103, Milford, MA) on an in-line 120 × 0.075 mm column using an UltiMate 3000 RSLCnano system (ThermoFisher Scientific, Waltham, MA; Cat#ULTIM3000RSLDNANO). Peptides were eluted using a 60 min gradient from 98:2 to 67:33 buffer A:B ratio. Eluted peptides were ionized by electrospray (2.25 kV) followed by mass spectrometric analysis on an Orbitrap Fusion Lumos mass spectrometer (ThermoFisher Scientific, Waltham, MA) using multi-notch MS3 parameters. MS data were acquired using the FTMS analyzer in top-speed profile mode at a resolution of 120 000 over a range of 375 to 1500 *m/z*. Following CID activation with normalized collision energy of 35.0, MS/MS data were acquired using the ion trap analyzer in centroid mode and normal mass range. Using synchronous precursor selection, up to 10 MS/MS precursors were selected for HCD activation with normalized collision energy of 65.0, followed by acquisition of MS3 reporter ion data using the FTMS analyzer in profile mode at a resolution of 50 000 over a range of 100–500 *m/z*.

Proteins were identified and TMT MS3 reporter ions quantified by searching the *Homo sapiens* database using MaxQuant (version 1.6.2.10, Max Planck Institute) with a parent ion tolerance of 3 ppm, a fragment ion tolerance of 0.5 Da, and a reporter ion tolerance of 0.001 Da. Protein identifications were accepted if they could be established with <1.0% false discovery and contained at least two identified peptides. Protein probabilities were assigned by the Protein Prophet algorithm (16).

TMT MS3 reporter ion intensity values were log2 transformed and missing values were imputed by a normal distribution for each sample using Perseus (Max Planck Institute). TMT batch effects were removed using ComBat (17) in order to correct for technical variation due to multiplexing samples across multiple TMT10plex batches. Statistical analysis was performed using Linear Models for Microarray Data (limma) with empirical Bayes (eBayes) smoothing to the standard errors (18). Proteins with an FDR adjusted *P*-value <0.05 and a fold change >2 were considered to be significant. Significant proteins were analyzed for protein networks and pathways using the Ensemble of Gene Set Enrichment Analyses (EGSEA) Bioconductor package (19) and Qiagen’s Ingenuity Pathway Analysis (20).

### Alkaline comet assay

This assay was conducted using the comet assay kit per manufactures instructions (Trevigen; Cat# 4252–040-K, Gaithersburg, MD). Briefly, 100 000 cells per condition were plated in a 12-well dish and pretreated for 24 h with either 680C91 (20 μM) or KYN (60 μM). Cells were treated with BCNU (125 μM) for 24 h before being harvested or let to recover for an additional 24 h ± 680C91/KYN. Harvested cells were washed in ice cold 1× PBS, and counted. Cells were diluted to 100 000 cells/ml and resuspended in LMAgarose to a final plated dilution of around 200–300 cells per well on the comet slide. The comet slide with wells coated with cell/agar mixture was incubated at 4°C in the dark to allow for the agar to solidify before immersing slide in proprietary lysis solution at 4 °C for 1 h. The comet slide was immersed in alkaline unwinding solution (200 mM NaOH and 1 mM EDTA in deionized H_2_O) for 20 min at RT before placing in the electrophoresis unit in ∼850 ml of alkaline electrophoresis solution (200 mM NaOH and 1 mM EDTA in deionized H_2_O) and running at 21 V for 30 min at 4°C. The comet slide was washed and dried completely at 37°C before staining with SYBR gold (Sigma Aldrich; Cat# S9430, St. Louis, MO) diluted in TE buffer (10 mM Tris-HCl pH 7.5, 1 mM EDTA). Comets were visualized using the EVOS microscope and scored using CometScore software. At least 190 comets were scored from at least two biological replicates, and tail moment was analyzed using one-way ANOVA with Tukey post-test.

### Wound migration assay

Cells were grown in a six-well dish until confluent and then pretreated with either 20 μM 680C91 or 60 μM KYN for 24 h. Cells were serum starved for 12 h prior to start of experiment. Wounds were created by manually scratching the surface of each well using a 1000 μl pipette tip. Wells were washed in 1× PBS to remove floating cell debris, and replaced with fresh media containing 680C91, KYN, and/or BCNU. Wells were imaged at 0, 2, 4, 8 and 10 h post scratch. At least 10 measurements of the wound diameter were taken at each timepoint, and average wound diameter over time was plotted to generate the wound migration rate. The experiment was completed in biological triplicate and a one-way ANOVA with a Tukey post-test was used to make comparisons between experimental conditions.

## RESULTS

### Inhibition of TDO leads to diminished resolution of RS induced by either HU or the bis-functional DNA damaging agent BCNU

**Figure 1.**
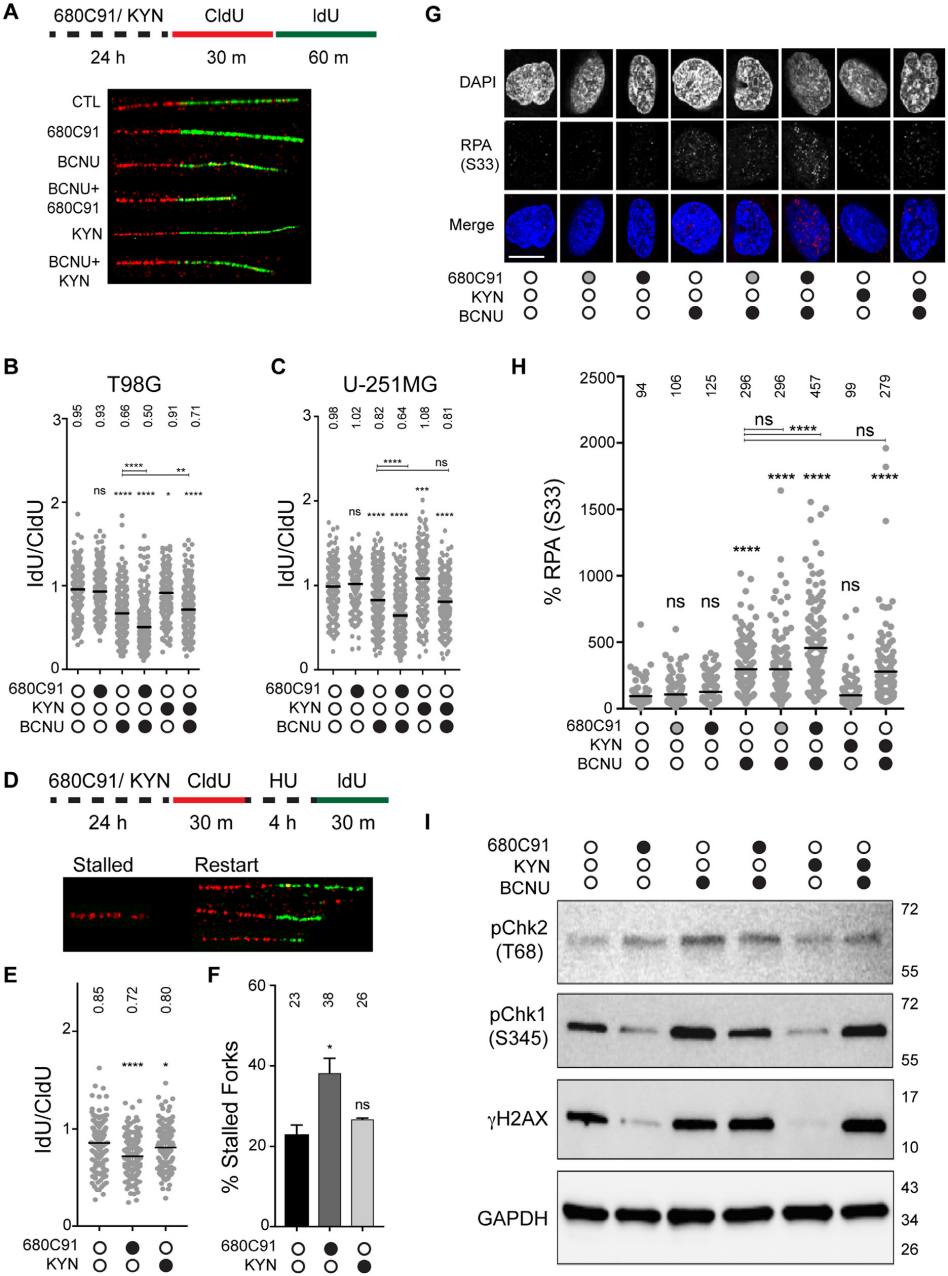
Inhibition of TDO leads to diminished resolution of stress at replication forks. (**A**) Cartoon schematic of analogue treatments and representative fiber images. Representative fibers were from experiments with T98G cells. (**B** and**C**) Quantification of IdU/CldU ratio for T98G and U-251 MG cells, where at least 290 fibers were scored from three biological replicates and mean ratio is depicted above each condition. CldU and IdU track lengths were normalized to individual pulse times prior to attaining IdU/CldU ratio. (**D**) Cartoon schematic and representative fiber images of stalled and restarted forks in T98G cells. (**E**) IdU/CldU ratio for restarted forks, where at least 179 fibers were scored from two biological replicates and mean ratio is depicted above each condition. For all DNA fiber spreading experiments, statistical significance was calculated using a Mann–Whitney *U* test, where * is *P*≤ 0.05, ** is *P*≤ 0.01, *** is *P* ≤ 0.001 and **** is *P* ≤ 0.0001. (**F**) Percentage of stalled forks after pre-treatment with 680C91 or KYN. The mean (± s.d.) is shown with the mean percentage of stalled forks for each condition is shown above the histogram. Statistical analysis was performed using a Student’s *t* test where * is *P* ≤ 0.05. (**G**) Representative immunofluorescence images of pS33-RPA2 in T98G cells +/- 680C91, KYN and BCNU; scale bar: 10 μm. Cells were treated with 680C91 (gray filled circle 10 μM; black filled circle 20 μM), KYN (60 μM), BCNU (125 μM) or a combination of these compounds. (**H**) Quantification of pS33-RPA2 fluorescence intensity relative to the control for the above-mentioned treatments, where at least 130 cells were quantified from three biological replicates. Normalized mean intensity values are depicted above each experimental condition. A one-way ANOVA with a Tukey post-test was used for statistical analysis, where * is *P* ≤ 0.05, ** is *P* ≤ 0.01, *** is *P* ≤ 0.001 and **** is *P* ≤ 0.0001. Asterisks shown above each condition are comparisons made relative to the control, asterisks shown above lines are for comparisons between the indicated conditions. (**I**) Immunoblot of cells treated +/- 680C91/ KYN/ BCNU, and probed for; pChk1 (Ser-345), pChk2 (Thr-68), γH2AX (Ser-139) and GAPDH.

Since gliomas (especially GBM) exhibit remarkably high levels of endogenous RS, we first examined the connection between KP signaling and replication dynamics. We investigated the effect of TDO inhibition on fork rate using the DNA fiber spreading (DFS) assay. We monitored DNA synthesis before exposure to BCNU (CldU, red tracks) and after BCNU treatment (IdU, green tracks; Figure 1A) in two glioblastoma-derived cell lines—T98G and U-251 MG (Figure 1B and C). A decrease in the ratio of IdU/CldU track lengths is indicative of fork slowing in response to treatment during the second pulse. We used a small-molecule inhibitor of TDO, 680C91 (or TDOi) to block KYN production. We and others have shown previously that 24 h incubation with the TDOi at micromolar concentrations reduces the amount of KYN in cell culture (10,14). The concentration of BCNU used in the DFS experiments (125 μM, 1 h) was sufficient to slow but not stop DNA synthesis, as determined by measuring EdU signal intensity (Supplementary Figure S4). By way of comparison, treating cells with HU (2 mM, 1 h) virtually eliminated all EdU signal (Supplementary Figure S4), indicative of complete cessation of DNA synthesis.

We first measured the impact of losing TDO activity on bypass of BCNU-induced DNA lesions. Treating glioma cells with the TDO inhibitor 680C91 for 24 h prior to the addition of IdU/CldU did not alter the rate of DNA synthesis (Figure 1B). As expected, treatment with BCNU during the second pulse (IdU) reduced the fork rate by ∼30% for T98G cells and ∼20% for U-251 MG cells (Figure 1B and C, please see figure legend for statistical analysis of changes discussed in the text). Inhibition of TDO activity enhanced the effect of BCNU on fork progression, as evidenced by another ∼15–20% decrease in the IdU/CldU ratio relative to treatment with BCNU alone for both cell lines (Figure 1B and C). We attempted to further modify fork rate by adding exogenous KYN (60 μM) to the cells. We selected 60 μM because KYN has been detected at micromolar concentrations in GBM cell culture (10,14,21). This is higher than physiological concentrations of KYN found in the brain, which have been estimated to lie in the nano- to low micromolar range (22). While addition of KYN alone did not impact fork progression in T98G cells, it did increase the elongation rate in U-251 MG cells by ∼10% (Figure 1C). When combined with BCNU, KYN protected against BCNU-mediated fork slowing in T98G cells but not U-251 MG cells (Figure 1B and C). Overall, these results are consistent with the idea that modulating KP signaling alters the capacity of T98G cells to effectively replicate DNA in the face of BCNU-induced damage—with TDO inhibition reducing bypass of BCNU-induced DNA damage.

We next determined if GBM-derived cells with attenuated KP could recover from hydroxyurea (HU)-induced RS (Figure 1D–F). We used a modified DFS protocol in which the CldU pulse is followed by treatment with HU (2 mM, 4 h), which stops DNA synthesis. After washing away HU, the ability to restart fork progress is estimated in two ways: (i) by measuring IdU track length and (ii) by calculating the fraction of tracks without IdU (stalled forks). Using this approach, we found that 680C91 pretreatment impaired fork restart (Figure 1E) and increased the fraction of forks stalled by HU treatment (Figure 1F). Pretreating cells with KYN did not alter fork recovery from HU-induced replication stress one way or another (Figure 1E and F). From these experiments, we concluded that T98G cells lacking TDO activity were more susceptible to HU-induced RS, consistent with the notion that KP signaling helps sustain an effective RSR in GBM-derived cells. With these results in hand, we went on to study the impact of TDO activity on markers of RSR and DDR programs.

### TDO activity is a determinant of RSR and DDR activation in T98G cells

We used immunofluorescence (IF) microscopy to monitor formation of phospho-S33 RPA2 (RPA32) in T98G cells. The serine 33 residue of RPA2 is one of the phosphatidylinositol 3-kinase related kinase (PIKK) consensus sites phosphorylated by the ATR kinase in response to replication fork stalling, facilitating stabilization of stalled forks and resolution of RS intermediates through the recruitment of factors, such as PALB2 and BRCA2, to sites of DNA damage/replication stress (23,24). In addition to pS33 RPA2, we also monitored changes in pS345 Chk1, pT68 Chk2 and γH2AX levels via immunoblotting. In this way, we hoped to discern whether TDO activity impacts ATR signaling either through Rad17-mediated Chk1 activation, which occurs in response to fork slowing/stalling or subsequent Nbs1-mediated ATR signaling and corresponds with accumulation of pS33 RPA2 and more extensive end-resection near collapsed forks (25). We used Chk2 phosphorylation and γH2AX as indicators of DNA break formation.

Treatment of cells with the TDO inhibitor 680C91 (10 or 20 μM for 24 h) did not alter the baseline signal intensity for pS33 RPA2 in GBM-derived T98G cells (Figure 1G and H). There was a notable decrease in pS345 Chk1 for cells treated with 680C91 (20 μM for 24 h), while pT68 Chk2 formation increased (Figure 1I). Similar to the RSR marker pChk1, the DNA damage marker γH2AX decreased following treatment with 680C91 (Figure 1I). The loss of pChk1 signal that accompanied TDO inhibition is interesting given that hpol κ has been shown to help activate the Rad17-arm of the ATR signaling cascade leading to phosphorylation of the Chk1 kinase. Previously we observed down-regulation of hpol κ in response to TDO inhibition (14). In this respect, the reduction in pS345 Chk1 when TDO was inhibited might be due to suppression of hpol κ/Rad17-mediated activation of ATR (25,26).

As expected, treating cells with BCNU (125 μM, 24 h) increased the pS33 RPA2 signal, consistent with an elevation in DNA damage-induced RS (Figure 1H). There was a concomitant increase in pChk1, pChk2 and γH2AX levels in response to BCNU treatment (Figure 1I). Pretreating cells with 10 μM 680C91 did not alter the level of pS33 RPA2 formed in response to BCNU treatment, but the addition of 20 μM 680C91 prior to BCNU exposure led to noticeably higher levels of pS33 RPA2 (Figure 1H), which could be interpreted as a diminished capacity for resolving BCNU-induced RS in cell lacking active KP signaling. Consistent with this notion, there was noticeable suppression of pChk1 activation when cells treated with 20 μM 680C91 were subsequently exposed to BCNU (Figure 1I). Activation of pChk2 and γH2AX formation following BCNU treatment was reduced slightly by TDO inhibition but not to the extent observed for pChk1 (Figure 1I). It is possible that TDO inhibition suppressed RSR activation through the hpol κ-Rad17 arm of ATR/Chk1 signaling, which led to an increased reliance on pS33 RPA2 accumulation and Nbs1-mediated ATR activation in response to BCNU-induced DNA damage. This is consistent with the clearly higher levels of pS33 RPA2 that coincide with noticeable reduction in pChk1 activation.

Adding exogenous KYN (60 μM, 24 h) did not change basal pS33 RPA2 levels in T98G cells (Figure 1H). The addition of KYN reduced pS345 Chk1 and γH2AX relative to DMSO but did not alter pT68 Chk2 levels (Figure 1I). When combined with BCNU treatment, exogenous KYN seemed to at least sustain Chk1 S345 phosphorylation and γH2AX levels, but the relative level of Chk2 activation in cells pretreated with KYN was kept below that observed for BCNU alone (Figure 1I). By way of comparison with 680C91, activating the KP with exogenous KYN seemed to maintain ATR/Chk1 signaling and limit ATM/Chk2 activation following treatment with BCNU whereas blockade of TDO activity shifted the damage response away from Chk1 arm of the ATR response—leading to an accumulation of pS33 RPA2. These findings support the idea that an active KP promotes the effective resolution of fork stress in glioma-derived cells.

### BCNU-induced nuclear γH2AX signal intensity was increased by TDO inhibition

**Figure 2.**
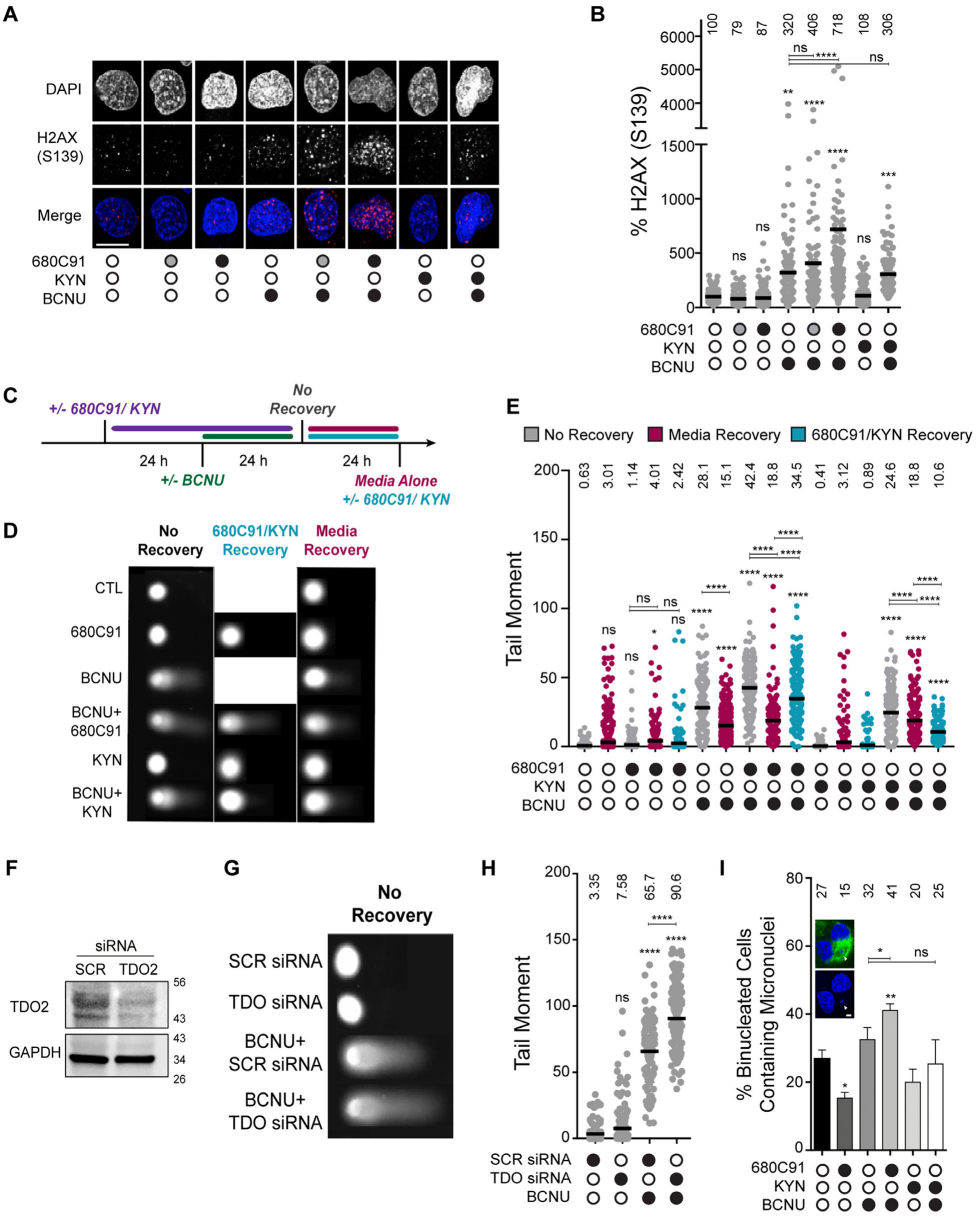
Loss of TDO activity results in delayed repair of BCNU-induced strand breaks and elevated chromosomal abnormalities. (**A**) Representative immunofluorescence images of γH2AX (pS139) in cells +/- 680C91, KYN and BCNU; scale bar: 10 μm. Cells were treated with 680C91 (gray filled circle 10 μM; black filled circle 20 μM), KYN (60 μM), BCNU (125 μM) or a combination of these compounds. (**B**) Quantification of γH2AX fluorescence intensity, where at least 160 cells were quantified from three biological replicates. The mean normalized intensity value for each condition is shown above the data points. (**C**) Cartoon of experimental scheme for monitoring DNA strand break formation with the alkaline comet assay. (**D**) Representative comet images of no recovery conditions or conditions in which treated cells were allowed to recover from BCNU in 680C91/KYN or in media alone. Cells were treated with 680C91 (20 μM), KYN (60 μM), BCNU (125 μM) or a combination of these compounds. (**E**) Tail moment for experiments outlined in panels (C and D), where at least two biological replicates were performed and at least 170 cells were scored. The mean tail moment value is shown above each experimental condition. (**F**) Immunoblot depicting knockdown of TDO2 via siRNA. (**G**) Representative comet images after siRNA knockdown of TDO2. SCR and TDO2 knockdown cells were treated with or without BCNU (125 μM) 24 h prior to comet assay. (**H**) Tail moment for experiments outlined in panel (G) where at least two biological replicates were performed and at least 104 cells were scored. The mean tail moment value is depicted above each experimental condition. (**I**) Representative image of a binucleated cell containing micronuclei, and quantification of the percentage of binucleated cells containing micronuclei. Cells were pretreated with 680C91 (20 μM) or KYN (60 μM) for 24 h prior to treatment with BCNU (125 μM) or media for another 24 h. The mean percentage of binucleated cells with MN is shown above the histogram for each experimental condition. At least three biological replicates were performed; scale bar: 5 μm. For all panels, statistical analysis was calculated using a one-way ANOVA with a Tukey post-test, where * is *P* ≤ 0.05, ** is *P* ≤ 0.01, *** is *P* ≤ 0.001 and **** is *P* ≤ 0.0001. Asterisks shown above each condition are comparisons made relative to the control, asterisks shown above lines are for comparisons between the indicated conditions.

Next, we investigated whether blockade of TDO activity had an effect on the level of nuclear γH2AX in GBM-derived T98G cells treated with BCNU. To more closely examine the relationship between TDO activity and γH2AX, we treated T98G cells with either 10 (gray circle) or 20 μM (black circle) 680C91 for 24 h and measured nuclear γH2AX signal intensity by IF microscopy (Figure 2A). Similar to our results with whole cell lysates, there was a decrease in nuclear γH2AX signal intensity in 680C91-treated cells, but at the selected concentrations the change was modest (Figure 2B).

We next tested for BCNU-induced changes in nuclear γH2AX. As expected, exposing cells to BCNU (125 μM, 24 h) increased the nuclear γH2AX signal detected by IF (Figure 2B). The combined effect of TDO inhibition and BCNU treatment led to a marked and dose-dependent increase in γH2AX signal intensity by IF microscopy (Figure 2B). The increase in γH2AX signal observed by IF was not as apparent from immunoblots with whole cell lysates. It is possible that this difference is due to ubiquitination of γH2AX, which could lead to species that were shifted on the Western blot but would be detected by IF. Adding KYN back to the cells did not have a major impact on nuclear γH2AX signal intensity (Figure 2B). When exogenous KYN was added prior to treatment with BCNU, there was not a detectable change in γH2AX by IF relative to BCNU alone (Figure 2B). In summary, the changes in γH2AX measured using IF microscopy were largely in-line with the 680C91-dependent alteration in pS33 RPA2 signal observed in response to BCNU treatment (Figure 1G), suggestive of an increase in unresolved DNA damage when TDO-deficient cells were exposed to BCNU.

### TDO inhibition slowed the rate of break repair following exposure to BCNU while addition of exogenous KYN enhanced break repair

Given the effects observed for replication dynamics and DDR activation, we next used the alkaline comet assay to determine if modulation of KP signaling led to altered levels of strand breaks (Figure 2C and D). We grew cells in the presence of DMSO (CTL), 680C91 (20 μM) or KYN (60 μM) for 24 h before adding BCNU (or DMSO) to the media for an additional 24 h and then measured DNA strand breaks (Figure 2C, ‘no recovery’). In the absence of DNA damage, culturing cells in either 680C91 or KYN alone did not have a significant impact on DNA strand breaks relative to the control (Figure 2E, results presented as gray data points).

Exposing cells to BCNU (125 μM, 24 h) increased the tail moment to 28.1, >40-fold over that observed for DMSO treated T98G cells (Figure 2E, gray data points for BCNU). Strikingly, pre-treatment with 680C91 increased the strand breaks induced by BCNU to 42.4, another 1.5-fold (∼50%) above that measured for BCNU alone (Figure 2E, gray data points for 680C91 + BCNU). Adding exogenous KYN to the cells prior to treatment with BCNU had a slight protective effect, as evidenced by a tail moment of 24.6 (Figure 2E, gray data points for 680C91 & KYN), which was less than the tail moment of 28.1 observed for BCNU alone. We repeated these experiments in an additional GBM-derived cell line (U-251 MG) and found a similar increase in tail moment after co-treatment with 680C91 and BCNU (Supplementary Figure S5).

The initial comet assay results led us to wonder if continued modulation of TDO activity and KP signaling would impact DNA repair once BCNU was removed. To investigate this possibility, we measured strand-break formation in cells treated as before except that we allowed an additional 24 h recovery period after BCNU was removed. Repair was allowed to proceed in media (DMSO/CTL), 680C91 (20 μM) or KYN (60 μM). In this way, we were able to determine if KP signaling impacted the kinetics of break repair.

First, we controlled for repair of endogenous strand-breaks by measuring the tail moment from cells grown for 48 h in the presence of DMSO (CTL), 680C91, or KYN and allowed to recover an additional 24 h in media. Allowing the DMSO-treated cells to grow an additional 24 hours in media increased the tail moment slightly from 0.63 to 3.01 (Figure 2E, compare gray control in the first column with the magenta data points in the second column). Culturing T98G cells for 48 h in the presence of 680C91 followed by a 24 recovery in media alone did not change the tail moment significantly compared to the DMSO-treated control (Figure 2E, compare a tail moment of 3.01 for the untreated control with a tail moment of 4.01 for 680C91). Cells grown for 48 h in the presence of exogenous KYN followed by a 24 recovery in media alone did not alter the tail moment relative to the DMSO-treated control (Figure 2E, compare a tail moment of 3.01 for the untreated control with a tail moment of 3.12 for KYN). We went on to examine the effect of KP signaling on repair of BCNU-induced DNA damage.

For cells treated with BCNU alone, the additional recovery period reduced the tail moment ∼45%, from 28.2 to 15.1 (Figure 2E, comparing BCNU alone in gray with BCNU alone + 24 h media recovery in magenta), indicative of active repair of BCNU-induced strand-breaks. For cells pretreated with the TDO inhibitor prior to BCNU, removing 680C91 and allowing cells to recover in media alone reduced the tail moment ∼55%, from 42.5 to 18.8 (Figure 2E, compare gray to magenta for BCNU + 680C91). Culturing GBM cells with exogenous KYN and BCNU, followed by recovery in media reduced the tail moment from 24.6 to 18.8 (Figure 2E, compare gray with magenta for BCNU + KYN). In short, cells treated with BCNU and allowed to recover in media for 24 h exhibited a reduction in the number of strand breaks, and this reduction was most pronounced for cells that had been exposed to the TDO inhibitor—perhaps indicative of a scenario where restoration in TDO activity following removal of 680C91 stimulated DNA repair.

Next, we allowed the cells to recover in the presence of either 680C91 or KYN. Allowing the cells to grow for an additional 24 h in the presence of 680C91 alone (i.e., no BCNU) did not change the tail moment much compared to recovery in media (Figure 2E, compare the tail moment of 4.01 for recovery in media to a tail moment of 2.42 for 72 h culture in the presence of 680C91, light blue data points). However, continuous inhibition of TDO during the recovery period prevented the efficient repair of BCNU-induced strand breaks, as evidenced by the fact that the tail moment decreased <20% from 42.5 to 34.6 (Figure 2E, compare gray to light blue for BCNU + 680C91). This was compared to the tail moment of 18.8 observed for cells exposed to BCNU and 680C91 then allowed to recover in media.

The effects on break repair for cells allowed to recover an additional 24 h in exogenous KYN were also interesting. Recall that there was a reduction in tail moment from 24.6 to 18.8 when cells exposed to exogenous KYN and BCNU were then allowed to recover in media (Figure 2E, right side of plot—compare gray to magenta for BCNU + KYN). When cells were allowed to recover in the presence of exogenous KYN, there was an even larger decrease in the tail moment (24.6 to 10.6; far-right side of Figure 2E—compare gray to light blue for BCNU + KYN). These results support the idea that excess KYN increased the rate of repair of BCNU-induced DNA strand breaks. This is in contrast to the delayed repair of DNA breaks observed when TDO activity was blocked.

To further confirm that results observed with 680C91 treatment were due to TDO inhibition and not potential off target effects, we used siRNA to knockdown expression of the *TDO2* gene (Figure 2F). Suppression of TDO at the protein level was verified by immunoblotting and cells were treated with BCNU, KYN or a combination of the two compounds (Figure 2G). A nontargeting siRNA pool (SCR) was used as a control. With TDO knockdown, we observed a slight but non-significant increase in tail moment relative to the SCR control (Figure 2H). As seen previously, tail moment increased dramatically after treatment of SCR cells with BCNU (3.35 to 65.7, Figure 2H). The amount of damaged DNA was further elevated after TDO knockdown (from 65.7 to 90.6 or an additional ∼38% relative to BCNU treatment alone) (Figure 2H). Pre-treatment with KYN prior to BCNU did not change the tail moment (compare 65.7 in BCNU alone to 63.2 in BCNU+ KYN). Taken together, these results were consistent with experiments performed with 680C91, supporting the notion that the sustained KP activity often observed in GBM could play a role in how these tumors repair and respond to genotoxic chemotherapeutics.

### BCNU-induced chromosomal damage was enhanced in cells with attenuated KP activity

To monitor the effect of KP signaling on chromosomal instability (CIN), we analyzed changes in MN. Treating T98G cells with 680C91 (20 μM, 24 h) reduced the number of binucleated cells with MN from 27% to 15% (Figure 2I), similar to what we reported previously with a lower dose of 680C91 (14). Treating cells with BCNU increased the percentage of cells with MN to 32%, as expected (Figure 2I). The percentage of binucleated cells with MN increased significantly to 41% when 680C91 treatment preceded exposure to BCNU (Figure 2I), in line with the comet assay results. The addition of exogenous KYN (60 μM, 24 h) reduced MN formation slightly compared to the DMSO control, although the *P*-value was >0.05 (Figure 2I). Similarly, adding KYN resulted in a slight (but nonsignificant) protection from BCNU-induced MN (Figure 2I, compare BCNU alone to BCNU + KYN). In summary, modulating KP signaling through inhibition of TDO had a notable impact on CIN in GBM-derived cells but the addition of exogenous KYN produced very little if any change in the formation of BCNU-induced CIN.

### KYN signaling alters progression through the S and G2/M checkpoints in BCNU-treated cells

**Figure 3.**
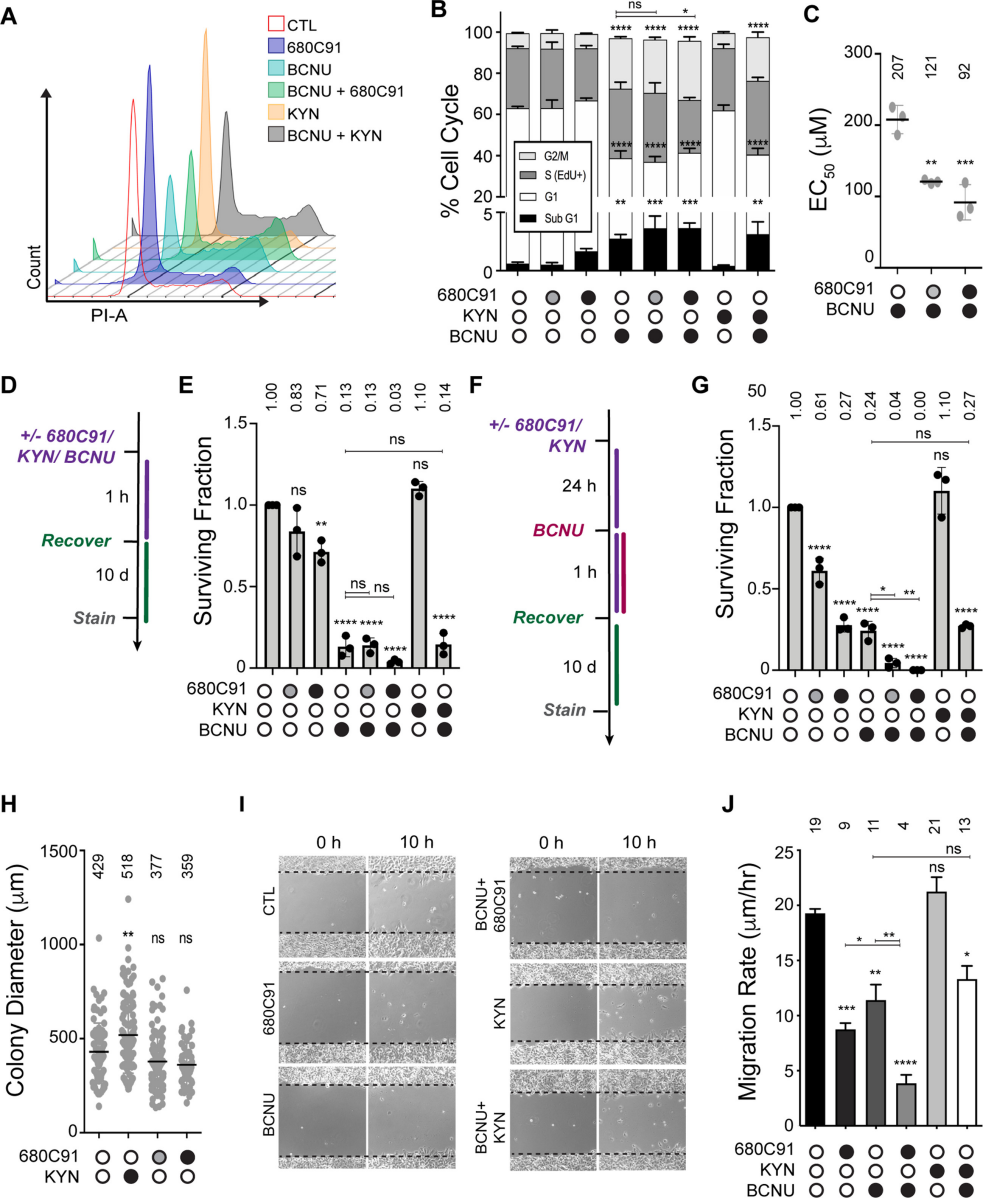
Inhibition of TDO sensitizes glioma cells to BCNU and reduces tumor cell migration. (**A**) Representative flow cytometry analysis of DNA content for selected experimental conditions depicted in the graph key. (**B**) Flow cytometry analysis of cell cycle changes occurring after 680C91/KYN/BCNU treatment and labeling with EdU/PI. (**C**) EC_50_ values calculated after dose response experiments were performed with varying concentrations of BCNU with either 10 (gray circle) or 20 μM (black circle) 680C91. (**D**) Treatment scheme for clonogenic assay, where cells were treated 680C91/KYN/BCNU for 1 h and allowed to recover for 10 d. Cells were treated with 680C91 (gray circle, 10 μM; black circle, 20 μM), KYN (60 μM), BCNU (125 μM) or a combination of these compounds. (**E**) Surviving-fraction calculated by counting colonies composed of at least 25 cells for experimental conditions described in panel (D). (**F**) Treatment scheme for clonogenic assay where cells were pretreated with 680C91/KYN 24 h prior to 1h BCNU treatment. As with the experiments in panels (D and E), cells were treated with 680C91 (gray circle, 10 μM; black circle, 20 μM), KYN (60 μM), BCNU (125 μM) or a combination of these compounds. (**G**) The surviving fraction was calculated by counting colonies composed of at least 25 cells for each experimental condition described in panel (F). (**H**) Relative colony diameter measured from all countable colonies for cells treated with 680C91 or KYN alone using conditions described in panel (F). (**I**) Representative wound migration images showing initial scratch and distance migrated at 34 h after scratch. Cells were treated with 680C91 (20 μM), KYN (60 μM), BCNU (125 μM) or a combination of these compounds. (**J**) Wound migration rate was calculated from changes in wound diameter over time for the experimental conditions described in panel (I). For all panels, error bars represent the s.d. calculated from three biological replicates. Statistical analysis was calculated using one-way ANOVA with Tukey post-test, where * is *P* ≤ 0.05, ** is *P* ≤ 0.01, *** is *P* ≤ 0.001 and **** is *P* ≤ 0.0001. Asterisks shown above each condition are comparisons made relative to the control, asterisks shown above lines are for comparisons between the indicated conditions.

We next sought to determine if combining KP modulation with DNA damage had an influence on cell cycle progression. Treating T98G cells with 10 μM 680C91 had minimal effect on cell cycle distribution, but treatment with 20 μM 680C91 exerted a modest anti-proliferative effect on T98G cells (Figure 3A, B and Supplementary Figure S1). At 20 μM 680C91, there was a slight decrease in the fraction of cells in S-phase (>2N)—from 29.3% for the untreated control to 25.4% for 680C91 treated cells—and a slight increase in the sub-G1 (<2N) population—from 0.6% for untreated cells to 1.7% for cells exposed to 20 μM 680C91 (Figure 3B).

BCNU treatment produced an increase in the S-phase (EdU^+^) population and an increase in the G2/M (4N) populations, indicative of delayed progression through S-phase, as well as G2/M arrest. The EdU^+^ population increased from 29.3% to 33.6% upon exposure to BCNU, whereas the G2/M population increased from 7.2% for the control to 24.5% for BCNU treated cells (Figure 3B). The sub-G1 fraction of cells also increased from 0.6% to 2.7% at this concentration of BCNU (Figure 3B).

Pre-treating cells with 10 μM 680C91 before exposure to BCNU increased the sub-G1 population from 2.7% for BCNU alone to 3.6% for BCNU + 10 μM 680C91 (Figure 3B). This increase corresponded with a small reduction in the 2N (G1) population of cells—from 36.1% for BCNU alone to 33.4% for BCNU + 10 μM 680C91, but this change was not considered statistically significant (*P*-value > 0.05). Pre-treating cells with 20 μM 680C91, on the other hand, led to a statistically significant increase in the G2/M population—from 24.5% for BCNU alone to 28.7% for BCNU + 20 μM 680C91—and a reduction in the EdU^+^ population—from 33.6% for BCNU alone to 25.6% for BCNU + 20 μM 680C91—that was also considered statistically significant (Figure 3B). The higher concentration of 680C91 also increased the sub-G1 population from 2.7% for BCNU alone to 3.6% for BNCU + 20 μM 680C91 (Figure 3B) but this change was not considered significant (*P*-value > 0.05). All in all, blocking TDO action corresponded with an enhancement of the antiproliferative effects induced by BCNU. Adding exogenous KYN (60 μM, 24 h) did not alter the cell cycle distribution of T98G cells to an extent that reached statistical significance (Figure 3A, B and Supplementary Figure S1). The overall effects of KP modulation on cell cycle distribution by either 680C91 or KYN were modest, even when BCNU treatment was added.

### Blocking TDO activity reduced the viability and proliferative capacity of glioma-derived cells treated with BCNU

We went on to measure KP-related changes to cell viability following BCNU treatment. We performed dose–response experiments to measure the effect of TDO inhibition on the viability of T98G cells treated with BCNU. Under the conditions used here, we measured an EC_50_ value of approximately 200 μM for T98G cells treated with BCNU alone (Figure 3C). Pre-treating cells with either 10 (gray circle) or 20 μM (black circle) 680C91 for 24 h reduced the EC_50_ value of BCNU to 125 and 100 μM, respectively (Figure 3C). The results obtained using the Calcein-AM assay were consistent with the notion that blocking KP signaling increased the cytotoxic effects of BCNU-induced DNA damage in GBM-derived cells.

We then examined the effect of KP modulation on the replicative capacity of T98G cells by measuring changes in clonogenic survival (Figure 3D). Treating 500 GBM-derived cells with either 10 μM or 20 μM 680C91 for 1 h reduced clonogenic survival 20% and 30%, respectively (Figure 3E). Treatment with BCNU (125 μM, 1 h) reduced colony formation almost 90% from that of the untreated control (Figure 3E). Co-treating T98G cells with BCNU and 10 μM 680C91 did not alter the proliferative capacity of T98G cells. However, the surviving fraction was reduced another 80% relative to BCNU alone when cells were co-treated with BCNU and 20 μM 680C91 (Figure 3E), indicative of synergy between BCNU-induced DNA damage and KP blockade in the impairment of glioma cell proliferation. Treatment with KYN alone (60 μM, 1 h) did not alter clonogenic survival either alone or when cells were co-treated with KYN and BCNU (Figure 3E).

Since the effects of TDO inhibition may not be apparent after a 1 h exposure to KP modulating agents, we next measured clonogenic survival for T98G cells pretreated with either 680C91 or KYN for 24 h. We observed a more pronounced decrease in colony formation when T98G cells were exposed to either 10 (gray circle) or 20 μM (black circle) 680C91 for the additional amount of time (Figure 3F). Treating cells with 10 μM 680C91 reduced the surviving fraction by ∼40%, while treatment with 20 μM 680C91 dropped the number of colonies to ∼25% of that observed for untreated cells (Figure 3G). Similar to the results with 1 h exposure, adding 125 μM BCNU for 1 h after culturing the cells for an additional 24 h in media reduced clonogenic survival ∼75% (compare BCNU alone for Figure 3E and G). In contrast to the results obtained with a 1 h co-treatment, a 24 h pre-treatment with 10 μM 680C91 enhanced the anti-proliferative effects of BCNU, reducing clonogenic survival to <20% of that observed for BCNU alone, and under the conditions tested here, we did not observe any surviving colonies when T98G cells were pretreated with 20 μM 680C91 for 24 h and then exposed to 125 μM BCNU for 1 h (Figure 3G), which is again indicative of an enhanced anti-proliferative effect for BCNU when KP signaling is suppressed.

As with the 1 h exposure, the addition of KYN alone (60 μM, 24 h) did not alter clonogenic survival when used alone or when cells were co-treated with KYN and BCNU (Figure 3G). We observed a robust increase in the average colony diameter for cells pretreated with exogenous KYN (Figure 3H). Although not considered statistically significant, there was a corresponding decrease in the average colony diameter for cells pretreated for 24 h with 680C91 (Figure 3H). In summary, the proliferation of GBM-derived cells over an extended period of time seem to be impacted by modulation of KP signaling and the anti-proliferative effects of BCNU were augmented by blockade of TDO activity.

### TDO inhibition impairs glioblastoma cell motility

Given the previously established role for the KP in promoting malignant properties of gliomas, we were curious to learn whether the combining inhibition of TDO with a genotoxin impacted tumor cell motility. To investigate this possibility, we measured T98G cell migration with the scratch-wound assay (Figure 3I). The rate of cell migration decreased from 19.3 μm/h for control cells to 8.7 μm/h when cells were exposed to 20 μM 680C91 (Figure 3J). BCNU (125 μM) treatment alone decreased migration rate to 11.4 μm/h, while co-treatment of BCNU and 680C91 decreased the migratory potential 2-fold relative to 680C91 alone and nearly 3-fold relative to BCNU treatment (Figure 3J). The addition of exogenous KYN did not have a substantial effect on T98G migration rate, increasing only slightly to 21.3 μm/h (Figure 3J). The results of the wound migration assay are consistent with the idea that the inhibition of TDO has a more pronounced impact on GBM cell motility than treatment with BCNU (at least under the conditions reported here) and that this effect can be potentiated with the co-treatment of BCNU and 680C91.

### Inhibition of TDO resulted in loss of sirtuin signaling and broad changes in genome maintenance, including depletion of 53BP1

**Figure 4.**
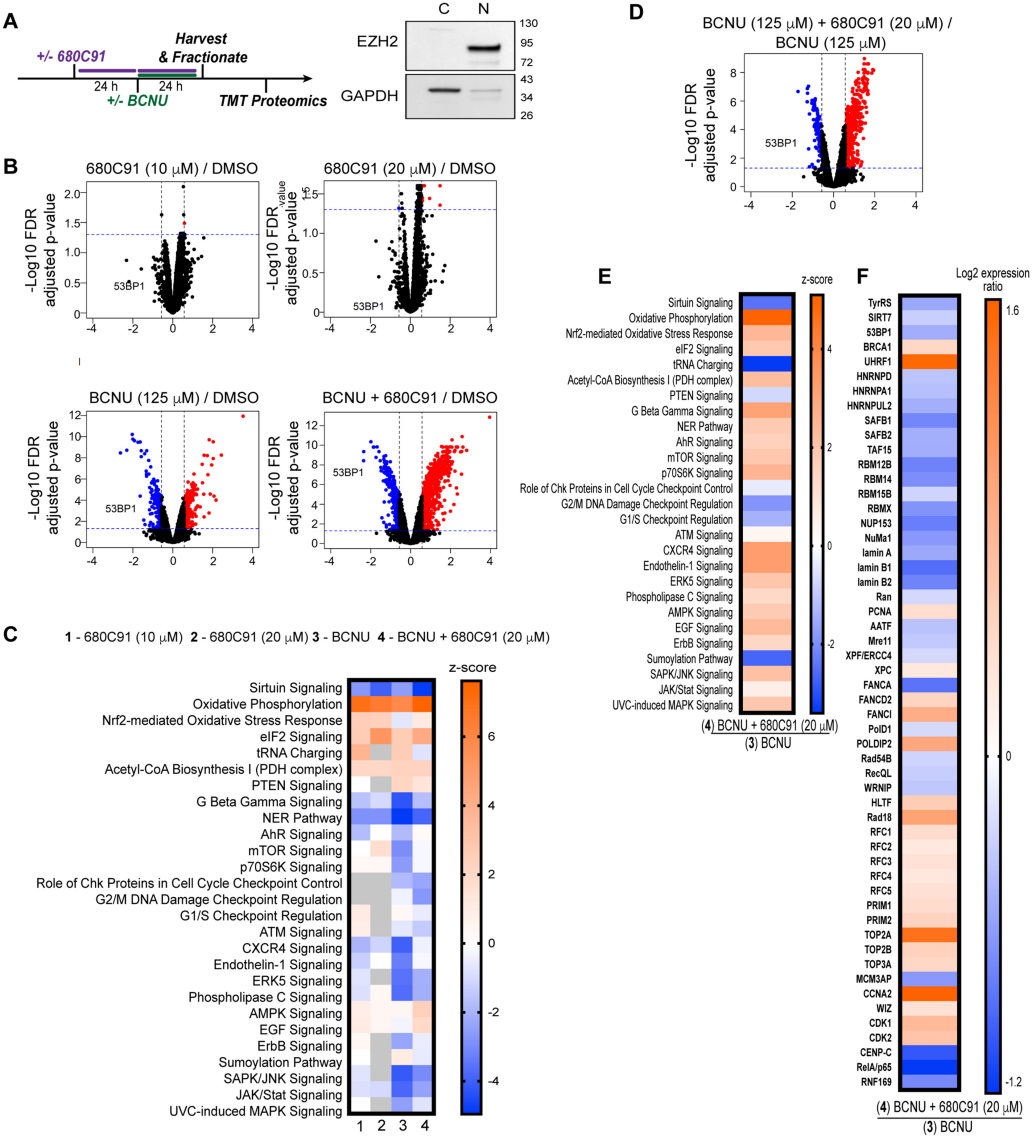
Sirtuin signaling, 53BP1, and other key DNA repair factors are reduced in glioma cells treated with the TDO inhibitor. (**A**) Cartoon scheme of experimental design for TMT proteomics experiment, and immunoblot depicting nuclear and cytoplasmic fractionation; where EZH2 was used as a nuclear marker and GAPDH was used as a cytoplasmic marker. (**B**) Volcano plots representing the limma log2 fold change and FDR adjusted *P*-value results from the following conditions: CTL versus 680C91 (10 and 20 μM), CTL versus BCNU and CTL versus BCNU+ 680C91. Proteins up-regulated in the first group of the comparison with at least a fold change >1.5 are highlighted in red while down-regulated proteins are in blue. Protein 53BP1 is highlighted in each of the different comparisons. The horizontal blue dotted line indicates a *P*-value threshold of 0.05 and the vertical black dotted lines indicate a fold change of 1.5 and -1.5. (**C**) Heat map of cellular pathway changes (*Z*-scores) predicted from comparison of the proteomic results for the following conditions to the DMSO control sample: (i) 680C91 (10 μM), (ii) 680C91 (20 μM), (iii) BCNU, (iv) BCNU+ 680C91 (20 μM) relative to the control. A *Z*-score of 2 or greater shows upregulation (orange), while -2 or less was considered downregulation of pathway activity (blue). (**D**) A volcano plot showing changes in protein abundance between the BCNU versus BCNU+ 680C91 (20 μM) conditions. The decrease in 53BP1 abundance is highlighted. (**E**) Heat map of cellular pathway comparison of the BCNU alone sample with the proteomic results obtained for the BCNU+ 680C91 (20 μM) sample. (**F**) Heat map showing Log2 fold-change in the abundance of individual proteins when comparing the BCNU alone sample with the BCNU+ 680C91 (20 μM) sample.

We next employed a quantitative mass spectrometric approach to identify proteomic changes associated with inhibition of TDO and exposure to BCNU (Figure 4). In an effort to focus on changes in DNA replication/repair factors, we enriched for the nuclear fraction from T98G cells treated with 680C91, BCNU, or a combination of both agents. We performed immunoblotting to confirm successful nuclear enrichment (Figure 4A). We then used the tandem-mass tag (TMT) isobaric labeling approach to quantify changes in abundance between experimental conditions. Each experimental condition was performed in biological quadruplicate. Mass spectrometric analysis was performed and a total of 5787 proteins were identified across all samples (Supplementary Table S2). Changes in protein abundance were considered significant if the FDR adjusted *P*-value was <0.05 in the respective sample groups. We used Qiagen™ Ingenuity Pathway Analysis (IPA) to guide our evaluation of the global changes in cellular pathways (Supplementary Tables S3–S8). We also performed manual inspection of the proteomic results to identify changes in individual proteins of interest. Comparing the treated samples to the control allowed us to identify several changes at the pathway level (Figure 4B and C), while comparing BCNU treatment alone to BCNU with 680C91 was also informative (Figure 4D). A complete description of our analysis of the proteomics results may be found in the supporting information, with additional details related to TDO-related changes located in Supplementary Figures S6 and S7. Our attention was drawn to two TDO-dependent changes in the proteome: the first change being related to 680C91-mediated depletion of 53BP1, a central node in regulation of the DDR, while the second change involved 680C91-mediated suppression of the NAD-dependent sirtuin SIRT7.

### Inhibition of TDO impairs 53BP1 recruitment to sites of DNA damage

**Figure 5.**
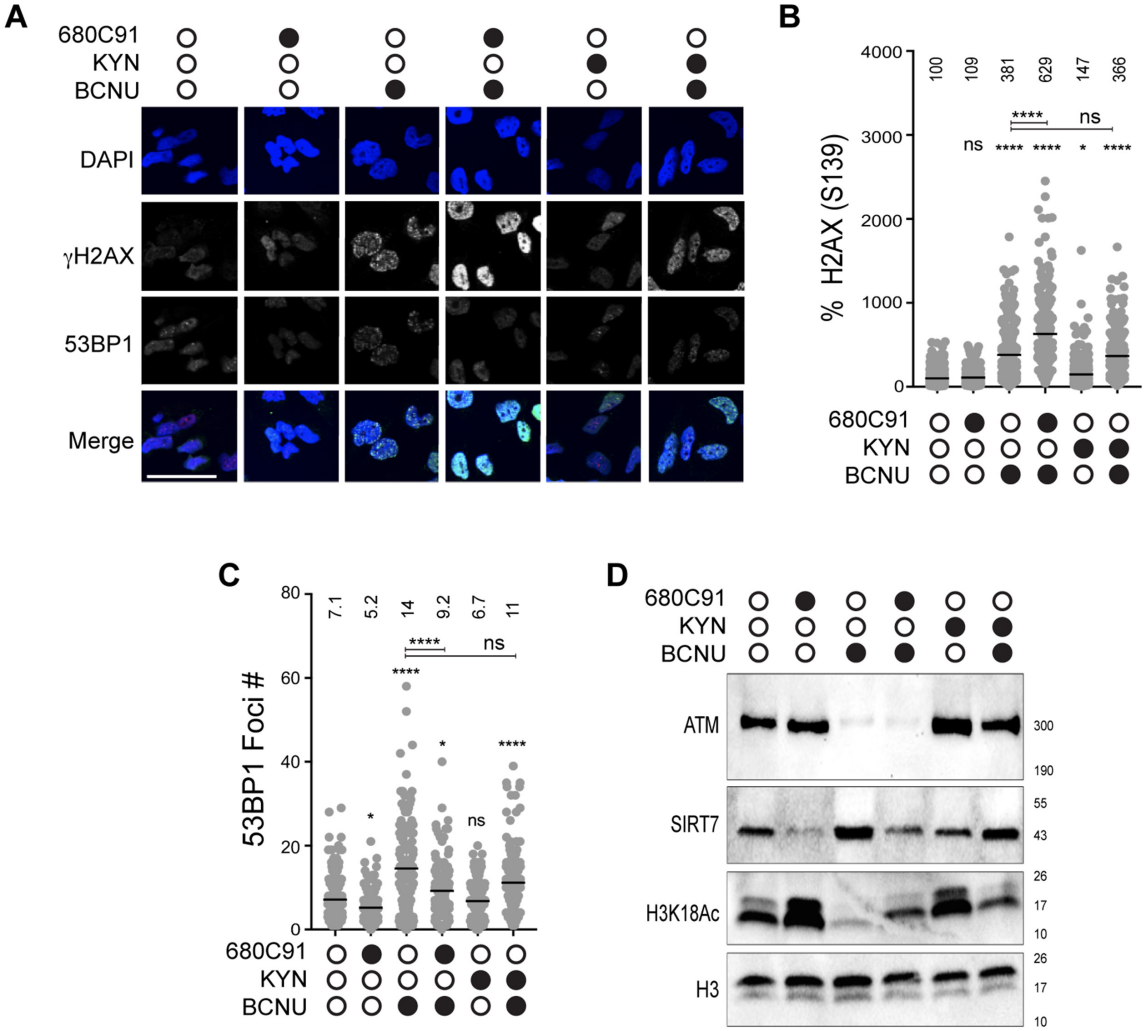
TDO inhibition reduces chromatin-bound SIRT7 and recruitment of 53BP1 to sites of BCNU-related damage. (**A**) Representative immunofluorescent images of γH2AX and 53BP1 stained cells after treatment +/- 680C91, KYN, BCNU; scale bar: 50 μm. (**B**) Plot of γH2AX normalized signal intensity. The mean intensity value is shown above the data points. (**C**) Graph of 53BP1 foci number per cell, where mean foci number is listed above each treatment. (**D**) Immunoblots are shown for chromatin bound ATM, ATR, SIRT7 and H3K18Ac. Histone H3 was used as a loading control. Cells were treated with 680C91 (20 μM), KYN (60 μM) and BCNU (125 μM) or a combination of these compounds as described in the ‘Materials and Methods’ section. For panels (B and C), at least 140 cells were quantified from two biological replicates. A one-way ANOVA was used for statistical analysis where * is *P* ≤ 0.05, ** is *P* ≤ 0.01, *** is *P* ≤ 0.001 and **** is *P* ≤ 0.0001. Asterisks shown above each condition are comparisons made relative to the control, asterisks shown above lines are for comparisons between the indicated conditions.

The results of the proteomics experiments allowed us to identify a number of changes in strand-break repair pathways that seemed to be regulated to some degree by TDO activity. A key finding from our proteomic analysis was that nuclear 53BP1 levels were diminished in cells co-treated with 680C91 and BCNU (Figure 4D and F). The 53BP1 protein serves as an important regulator of the partitioning between HR and NHEJ double-strand break repair pathways—with high-levels of 53BP1 favoring NHEJ over HR. We postulated that the sustained levels of BCNU-induced breaks observed with the comet assay for cells treated with the TDO inhibitor might be related to defective recruitment of 53BP1 to sites of DNA damage. To test this idea and help validate our proteomic results, we measured changes in 53BP1 foci formation via IF microscopy. Pre-extraction of cytosolic proteins and co-staining for DAPI ensured that the signal was from chromatin-bound 53BP1 (Figure 5A). Chromatin bound γH2AX was used as a proxy for how much DNA damage signal was present. We quantified foci formation for 53BP1 but the pan-nuclear signal observed for some conditions prevented an accurate assessment of γH2AX foci. For that reason, we report γH2AX signal intensity per cell. The experimental design was identical to that used for the comet assay, with cells being exposed to treatment with DMSO, 680C91 (20 μM), or KYN (60 μM) for 24 h prior to co-treatment with BCNU for another 24 h.

In contrast to the reduction in γH2AX measured by immunoblotting and IF microscopy of total nuclear protein (Figures 1 and 2), there was effectively no change in chromatin-bound γH2AX signal intensity for cells exposed to 680C91 (Figure 5A and B). Treatment with 680C91 alone reduced the number of 53BP1 foci ∼15% relative to control (Figure 5A and C). As expected, the addition of BCNU increased the chromatin-bound γH2AX signal >3-fold (Figure 5B) and 53BP1 foci ∼2-fold (Figure 5C), indicative of DDR activation. Pre-treating cells with 680C91 led to diminished 53BP1 foci formation in response to BCNU exposure (Figure 5C) in spite of the fact that γH2AX signal intensity soared ∼40% above the value measured for cells treated with BCNU alone (Figure 5B). These results validated an important aspect of our proteomics experiments—loss of TDO activity impairs the ability of GBM cells to successfully recruit 53BP1 to sites of DNA damage.

Treating cells with exogenous KYN produced a small increase the γH2AX signal relative to control (Figure 5B) but did not change the basal level of 53BP1 foci (Figure 5C). Combining KYN with BCNU reduced 53BP1 foci relative to BCNU alone (Figure 5C), but there was also a slight reduction in γH2AX signal intensity for KYN-treated cells exposed to BCNU compared to treatment with BCNU alone (Figure 5B). In other words, adding exogenous KYN reduced the amount of BCNU-induced γH2AX signal (likely through more efficient repair based on the comet assay results), which may have expedited removal of chromatin-bound 53BP1. By way of comparison, combining BCNU treatment with TDO inhibition led to higher γH2AX signal, fewer 53BP1 foci, and increased strand break formation than cells treated with BCNU alone.

### Chromatin-bound ATM and SIRT7 are altered by changes in KP signaling

Once we had confirmed TDO-mediated effects on 53BP1 recruitment to chromatin, we turned our attention back to SIRT7 as a potential regulator of this process. Previously, others have shown that SIRT7 activity promotes 53BP1 recruitment through deacetylation of histone H3K18 (27). One study reported that SIRT7-catalyzed deacetylation was required to dissociate ATM from chromatin and completion of DSBR (28). Failure to deacetylate ATM led to increased levels of chromatin-bound 53BP1 and RPA2 (28). Given that SIRT7 was identified in our proteomics analysis as being altered by modulation of the KP, we decided to investigate whether the impaired recruitment of 53BP1 could be related to changes in SIRT7 action (Figure 5D).

First, we probed for KP-dependent changes in chromatin-bound SIRT7 and ATM. A sharp decrease in chromatin-bound SIRT7 was observed for GBM cells treated with 680C91 (Figure 5D). The decrease in SIRT7 produced by treatment with 680C91 was accompanied by an increase in H3K18Ac but very little change in ATM bound to chromatin (Figure 5D). Again, the inverse correlation between SIRT7 localization and H3K18Ac was expected due to loss of the deacetylase action of SIRT7 on chromatin. These results were consistent with our proteomics analysis and the idea that TDO inhibition exerts downstream effects on SIRT7 localization and activity.

Comparing BCNU exposure with or without TDO inhibition further supported our working hypothesis, as blockade of TDO activity impaired SIRT7 recruitment to DNA following exposure to BCNU relative to treatment with the genotoxin alone (Figure 5D). H3K18Ac dropped sharply with BCNU treatment, consistent with more chromatin-bound SIRT7 (Figure 5D). Pre-treatment with 680C91 increased the amount of H3K18Ac present following exposure to BCNU—consistent with a reduction in chromatin-bound SIRT7 compared to cells treated with BCNU alone. The amount of chromatin-bound ATM was sharply reduced for cells treated with BCNU whether TDO was inhibited or not (Figure 5D).

The addition of exogenous KYN increased the amount of ATM bound to DNA without much of an effect on SIRT7 levels relative to DMSO-treated cells (Figure 5D). Addition of KYN did not seem to increase SIRT7 activity, as the amount of H3K18Ac was at least as much as DMSO-treated cells. Adding exogenous KYN led to a dramatic increase in chromatin-bound ATM following exposure to BCNU (Figure 5D, compare ATM signal for lane 3, BCNU alone to lane 6, KYN + BCNU). SIRT7 remained bound to chromatin when KYN-fed cells were exposed to BCNU (Figure 5D)—contrasting sharply with the depletion of SIRT7 that accompanied TDO inhibition. The activity of SIRT7 in cells treated with KYN and BCNU seemed to be slightly less robust than that observed for treatment with BCNU alone, based on H3K18Ac levels (Figure 5D), but H3K18Ac was reduced in cells treated with KYN and BCNU relative to treatment with KYN alone (Figure 5D, compare last two lanes for H3K18Ac blot). In summary, these experiments supported the notion that inhibition of TDO produced changes in SIRT7 activity on DNA that likely influenced the dynamics of break repair, including ATM and 53BP1 recruitment to sites of damage. The increase in chromatin-bound ATM afforded by exogenous KYN matches nicely with the faster kinetics of break repair observed with the comet assay. The relative increase in chromatin-bound SIRT7 for cells with an adequate KYN supply may allow for more effective shuttling of ATM and completion of DSBR than what occurs in GBM cells lacking TDO activity.

### TDO inhibition delays repair of IR-induced damage without altering 53BP1 foci formation

**Figure 6.**
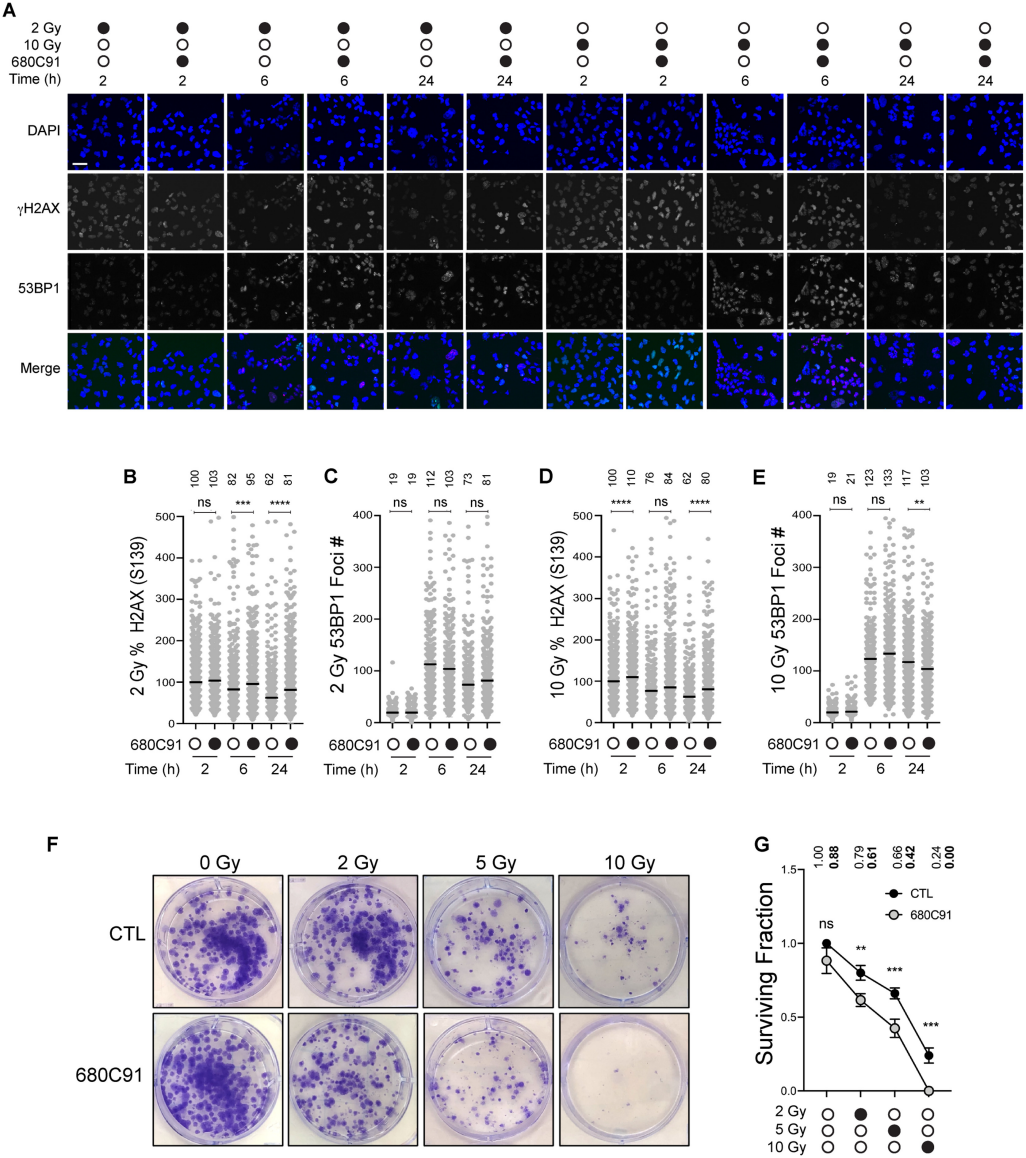
TDO inhibition delays repair of IR induced damage without altering 53BP1 recruitment. (**A**) Representative immunofluorescence images of T98G cells pretreated ± 680C91 prior to irradiation (2 or 10 Gy). Images depict cells at 2, 6 and 24 h post irradiation; scale bar = 50 μm. At least 300 cells were quantified from two biological replicates for all conditions reported in panels (B–E). (**B**) Plot of the percent γH2AX intensity relative to the average fluorescence intensity for the untreated cells (2 h) exposed to 2 Gy radiation. (**C**) Plot of 53BP1 foci number per cell for cells exposed to 2 Gy radiation. (**D**) Plot of the percent γH2AX intensity relative to the average fluorescence intensity for the untreated cells (2 h) exposed to 10 Gy radiation. (**E**) Plot of 53BP1 foci number per cell for cells exposed to 10 Gy radiation. (**F**) Representative images of colonies for cells exposed to 0, 2, 5 or 10 Gy IR (± 24 h pretreatment with 20 μM 680C91). (**G**) Graph of surviving fraction after exposure to irradiation alone (black circles) or pre-treatment with 680C91 for 24 h prior to IR (gray circles). For each experimental condition, three biological replicates were scored, where a colony was counted if it consisted of at least 25 cells. Mean values are shown above the data for all quantified experimental results (panels B–E and G). A one-way ANOVA was used for statistical analysis where * is *P* ≤ 0.05, ** is *P* ≤ 0.01, *** is *P* ≤ 0.001 and **** is *P* ≤ 0.0001.

The genotoxicity of BCNU is due in large part to replication-associated DNA damage. We wanted to determine if the TDO-dependent effects we observed with BCNU were applicable to a different source of DNA damage. We exposed GBM cells (± pre-treatment with 680C91) to either 2 or 10 Gy IR and allowed them to recover for 2, 6 and 24 h (Figure 6A). To assess DDR activation, we monitored changes in chromatin bound 53BP1 and γH2AX (Figure 6B–E). In this way, we were able to monitor both the initial formation of DNA damage (γH2AX) at 2 h and the kinetics of repair at 6 and 24 h.

TDO inhibition did result in significantly higher levels of γH2AX signal for both doses of IR (Figure 6B and D), although the relative magnitude of this change was not as large as that measured for BCNU-induced damage. Notably, the TDO-dependent elevation of γH2AX signal persisted over the duration of the time course, and at 2 Gy, the difference grew larger as time post-IR increased (Figure 6B). In contrast to what was observed with BCNU, the number of IR-related 53BP1 foci did not change much as a function of TDO inhibition. TDO inhibition did produce a statistically significant reduction in 53BP1 foci relative to the control 24 h after 10 Gy IR (Figure 6E), but again, these TDO-related changes were not nearly as dramatic as those observed for BCNU. Based on γH2AX signal, TDO inhibition did seem to impact the repair of IR-induced damage, but this effect did not correlate with changes in 53BP1 recruitment.

We next examined the impact of TDO inhibition on cell proliferation after IR treatment via clonogenic survival assay. We pretreated cells with 680C91 24 h prior to irradiation. Cells were either mock-treated or exposed to a single dose of 2, 5 or 10 Gy IR. Cells were then replated at low density and allowed to recover for 12 days prior to staining (Figure 6F). The surviving fraction was calculated and plotted as a function of IR dose (Figure 6G). There was a significant reduction in the surviving fraction at all three doses of IR. For the control cells, the surviving fraction decreased 21%, 34% and 76% at 2, 5 and 10 Gy, respectively (Figure 6G). For cells pretreated with the TDOi, the surviving fraction decreased 31% and 52% at 2 and 5 Gy (relative to the surviving fraction for TDOi alone), while virtually no colonies formed at 10 Gy (Figure 6G). The summation of these experiments supports a model where inhibition of TDO activity attenuates resolution of strand-break repair, as evidenced by the slower removal of γH2AX. The delay in break repair seemed to occur through a mechanism that did not involve altered 53BP1 recruitment to chromatin. The end-result of TDO inhibition was to sensitize T98G cells to IR.

## DISCUSSION

Studying factors that influence the robust RSR and DNA repair capacity of gliomas is an important part of understanding the etiology of the disease and for developing new, more effective treatment strategies. We have investigated the relationship between TDO activity and the maintenance of genomic integrity in glioma-derived cells—uncovering evidence that KP signaling has a broad effect on the ability of glioma-derived cells to respond to RS and DNA damage generated by BCNU, a DNA alkylating agent used in the treatment of malignant brain tumors. Additional effects related to recovery from HU-induced fork stalling and IR-related strand-breaks were also identified. The impact of KP signaling on central nervous system disorders, immune function and tumor biology is known to be multi-faceted, involving interplay between transcriptional regulators, NAD^+^-dependent activities, mitochondrial function, heme biosynthesis and energy utilization circuits (22, 29–32). While inferences drawn from our findings are limited by the *in vitro* nature of experiments, they provide some new and intriguing insights into how metabolic changes in the tumor microenvironment might influence genomic stability in glioma cells and possibly influence responsiveness to chemotherapy.

**Figure 7.**
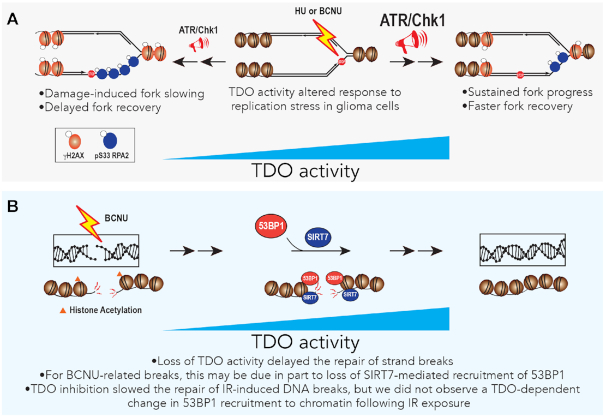
TDO activity impacts the response to replication stress and influences DNA repair in glioblastoma cells. (**A**) A model is shown illustrating the proposed effects of TDO activity on the RSR. Suppression of TDO activity via 680C91 treatment led to a reduced replication rate following BCNU treatment and delayed fork recovery following HU treatment. (**B**) A model depicting the impact of TDO activity on repair of DNA breaks is shown. An active KP, fueled by TDO activity, aids in the recruitment of 53BP1 to sites of BCNU-induced DNA damage, in part, through SIRT7-mediated deacetylation of histone H3K18. Inhibition of TDO impaired SIRT7 and 53BP1 recruitment to chromatin and resulted in delayed break repair, while stimulation of the KP with exogenous KYN increased the rate of break repair. TDO inhibition also delayed resolution of DNA damage marked by γH2AX and reduced clonogenic survival following exposure to IR, indicative of a broad role for TDO in stimulating timely repair of DNA damage. The role of TDO in governing 53BP1 localization may be specific to replication blocks or certain sources of DNA damage, as TDO inhibition did not have a major impact 53BP1 foci formation in response to IR-induced strand-breaks.

Our initial focus on DNA replication dynamics allowed us to explore the idea that TDO activity could influence fork progress and DNA damage tolerance by modulating the RSR. Analysis of replication rates using the DFS assay revealed that loss of TDO activity led to impaired fork progress following treatment with BCNU and less effective fork restart in response to HU treatment. The defects in fork restart could be related to the fact that TDO inhibition diminished the basal levels of some RSR factors, such as hpol κ and phosphorylated Chk1. While there are some inconsistencies in the literature, there is reasonable evidence to suggest that hpol κ can directly influence Rad17 and 9–1-1 complex-mediated recruitment to sites of replication stress (33). In line with this notion, pS345 Chk1 levels were reduced in T98G cells treated with 680C91 and there was less robust checkpoint activation in cells co-treated with 680C91 and BCNU. The reduced checkpoint activation was in contrast to the strong induction in pS33 RPA2 signal and increased G2/M arrest observed for cells co-treated with 680C91 and BCNU. These results may be indicative of increased replication-associated DSBs, as Nbs1-dependent phosphorylation of RPA2 recruits the MRN complex to perform extensive end-resection near sites of RS (25). A recent study found that hpol κ protects stalled forks from Mre11 nuclease activity to promote fork recovery (34). It is possible TDO inhibition in glioma cells leads to down-regulation of hpol κ, which then results in both (i) diminished Chk1 activation and (ii) increased resection by the MRN complex. In this regard, the KP could help control the hpol κ-Rad17 arm of the RSR. Disruption of this circuit may lead to a greater dependence on Nbs1-mediated recovery of collapsed forks. A less efficient response to DNA adducts blocking the fork would also explain slowing of the replication machinery and elevated pS33 RPA2 levels in response to BCNU-induced DNA damage. Experiments are ongoing exploring the role of hpol κ in the regulation of replication dynamics in gliomas. While there are many important mechanistic features yet to be discerned, the summation of our results implicates the KP in the resolution of RS inherent to GBM (Figure 7A).

In addition to changes in DNA replication dynamics, we also observed KP-dependent modulation of DNA break repair—with TDO inhibition delaying repair of BCNU-induced strand breaks and the addition of exogenous KYN promoting more rapid clearance of the damage. Recruitment of 53BP1 emerged from our proteomic analysis as a key node in the relationship between TDO activity and DNA repair. This finding was validated by monitoring TDO-dependent changes in 53BP1 foci. The mechanistic features that underlie TDO-dependent changes in 53BP1 dynamics are undoubtedly complex and involve multiple elements (e.g. LCD proteins, trafficking proteins like NUMA1 and NUP153, as well as other proteins directly involved in DNA repair). Of these factors, we explored the notion that impaired SIRT7 activity and localization coincided with defective recruitment of 53BP1 to sites of damage.

SIRT7 participates in multiple aspects of the DDR including chromatin changes and recruitment of DNA repair factors, and loss of SIRT7 sensitizes cells to multiple genotoxic agents (35–37). SIRT7 is itself recruited to DNA damage sites in a PARP-dependent manner and promotes NHEJ through H3K18 deacetylation and 53BP1 recruitment (27). Furthermore, hyperacetylated p53 accumulates in cells lacking SIRT7, resulting in apoptosis (38). SIRT7 also serves to protect against cell death from persistent DDR by deacetylating ATM late in the DSBR process with failed deacetylation of ATM promoting retention of γH2AX and apoptosis or senescence (28). Based on the results reported here, as well as previously published studies, we propose that activation of the KP in gliomas facilitates SIRT7-mediated recruitment of 53BP1 and subsequent repair of replication-associated strand breaks (Figure 7B). The recruitment of 53BP1 involves multiple players, but it is determined in part by SIRT7-catalyzed deacetylation of H3K18. Blockade of TDO action inhibits this pathway, delaying repair of breaks and rendering GBM cells more susceptible to BCNU-induced DNA damage. Our study builds upon previous findings to establish a functional link between KP activity in GBM cells and SIRT7-mediated effects on DNA repair—a link that could be related to changes in NAD^+^ supply.

In recent years, the far-reaching impact of NAD^+^-dependent processes on DNA damage, mitochondrial function, neurological disorders and organismal longevity has received much attention (39–41). Other work has highlighted the role of NAD^+^ levels in resistance to genotoxic anticancer drugs. For example, inhibition of the NAD^+^ salvage pathway sensitized the glioblastoma-derived LN428 cell line to TMZ when the nicotinamide phosphoribosyl transferase (NAMPT) inhibitor FK866 was combined with the BER inhibitor methoxyamine (MX) (42). Our results implicate *de novo* NAD^+^ synthesis from tryptophan in a similar phenomenon. Along these same lines, synthesis of NAD^+^ from the tryptophan catabolite QA was also shown to fuel protection against H_2_O_2_, TMZ and IR (43). The formation of QA depends on expression of several enzymes including quinolinate phosphoribosyltransferase (QPRT) activity in tumor cells and 3-hydroxy-anthranilic acid oxygenase (3-HAO), which is normally only expressed in microglial cells. QPRT expression is abnormally high in GBM patients, which may contribute to an increased reliance on *de novo* NAD^+^ supplies for genome protection. Interestingly, T98G cells were the only established cell line shown in a previous report to express 3-HAO (43), consistent with the augmented effects of BCNU we observed when TDO activity was inhibited.

To determine if the TDO-related effects on DNA repair observed with BCNU were broadly applicable to other sources of strand breaks, we monitored γH2AX/53BP1 dynamics and measured clonogenic survival in response to IR. As with BCNU, TDO inhibition did indeed delay resolution of IR-induced damage, as evidenced by elevated γH2AX signal, but this occurred without altered 53BP1 recruitment, indicative of distinct TDO-related impacts on the response to BCNU-related DNA damage and IR-induced strand breaks. Pre-treatment with the TDOi did sensitize cells to IR, with significant reductions in clonogenic survival at all three doses of IR (Figure 6G). These results are in-line with the sustained γH2AX signal we observed for T98G cells pretreated with the TDOi prior to IR exposure (Figure 6B and D) and lead us to the overall conclusion that loss of TDO activity delays repair IR-induced DNA damage and that this reduces the replicative capacity of the cells. At this point, it is unclear to us why TDO activity exerts an effect on 53BP1 localization in response to BCNU treatment but not IR-induced damage. Understanding the mechanistic basis for this difference will require additional study.

In conclusion, our finding that inhibition of TDO resulted in failed resolution of RS and delays in repair of BCNU- and IR-induced DNA damage carries important implications for how aberrant KP signaling might influence disease progression in gliomas through increased genomic instability and tolerance of therapy-induced DNA damage. Tumors re-wired to express high levels of TDO could have an elevated capacity for tolerating replication stress and DNA damage. The result of this phenomenon might include higher rates of mutagenesis and increased tumor heterogeneity, as well as an enhanced ability to survive genotoxic treatments. Additional work is needed to decipher the exact mechanisms promoting fork recovery and DNA repair in GBM cells with high TDO activity.

## DATA AVAILABILITY

All source data are stored on a secure UAMS server and can be made available upon request. Proteomics data are available via ProteomeXchange with identifier PXD022351. Flow cytometry data are available via Flow Repository with identifier FR-FCM-Z33X.

## Supplementary Material

zcab014_Supplemental_Files
