# Supplementary material for: Inhibition of tryptophan 2,3-dioxygenase impairs DNA damage tolerance and repair in glioma cells
Source: NAR Cancer. 2021 Apr 9;3(2):zcab014. doi: 10.1093/narcan/zcab014 (PMC8034706; doi:10.1093/narcan/zcab014)
Supplement: zcab014_Supplemental_Files [file zcab014_supplemental_files.zip › 210315 REED NAR Cancer SI.docx]

# Supplementary Materials for

Inhibition of tryptophan 2,3-dioxygenase impairs DNA damage tolerance and repair in glioma cells

Megan R. Reed^1^, Leena Maddukuri^1^, Amit Ketkar^1^, Stephanie D. Byrum^1,2^, Maroof K. Zafar^1^, April C. L. Bostian^1^, Alan J. Tackett^1,2^ and Robert L. Eoff^1^

From the ^1^Department of Biochemistry and Molecular Biology, University of Arkansas for Medical Sciences, Little Rock, AR 72205, U.S.A. and ^2^Arkansas Children’s Research Institute, 1 Children’s Way, Little Rock, AR 72202, U.S.A.

Address correspondence to:

Robert L. Eoff

Department of Biochemistry & Molecular Biology

University of Arkansas for Medical Sciences

325 Jack Stephens Drive

Little Rock, Arkansas 72205-7199

Telephone: (501) 686-8343

Fax: (501) 686-8169

E-mail: [RLEOFF@UAMS.EDU](mailto:martin.egli@vanderbilt.edu)

# *Running Title: Kynurenine signaling and genome maintenance in gliomas*

**Table of contents**

**Detailed description of proteomics analysis.**

**TABLE S1. Key resource and reagent list.**

**TABLE S2. List of proteins identified by TMT mass spectrometry across all samples.**

**TABLE S3. IPA comparing proteomic results for T98G cells treated with either DMSO or 10 μM 680C91.**

**TABLE S4. IPA comparing proteomic results for T98G cells treated with either DMSO or 20 μM 680C91.**

**TABLE S5. IPA comparing proteomic results for T98G cells treated with either DMSO or 125 μM BCNU.**

**TABLE S6. IPA comparing proteomic results for T98G cells treated with either 125 μM BCNU or 20 μM 680C91.**

**TABLE S7. IPA comparing proteomic results for T98G cells treated with either DMSO or 125 μM BCNU and 20 μM 680C91.**

**TABLE S8. IPA comparing proteomic results for T98G cells treated with either 125 μM BCNU alone or 125 μM BCNU and 20 μM 680C91.**

**FIGURE S1. Representative flow-cytometry results.**

**Figure S2. Uncropped immunoblots for whole cell lysate (WCL) samples.**

**Figure S3. Uncropped immunoblots for chromatin-bound (CB) samples.**

**FIGURE S4. T98G EdU incorporation immunofluorescence experiment.**

**FIGURE S5. Representative comet images and results from comet assay with U-251MG cells.**

**FIGURE S6. Immunoblotting revealed that TDO inhibition resulted in decreased deacetylase activity, PAR-ylation, and phospho-AKT levels.**

**FIGURE S7. Quantification of changes in protein abundance as a function of molecular weight.**

**Detailed Description of Proteomics Analysis.**

We compared the difference between DMSO treated cells and cells exposed to 680C91 (10 and 20 μM, 24 h). Overall changes in abundance were modest for individual proteins in both treatment conditions (**Fig. 4B**), but there were some interesting trends identified at the pathway level (**Fig. 4C**). AhR signaling was predicted to be diminished by treatment with 10 μM 680C91 (**Table S3**) but the change at the pathway level was ambiguous for cells treated with 20 μM 680C91 (**Table S4**). Both concentrations of 680C91 led to a reduction in sirtuin signaling (10 μM: z-score = -2.71, p-value = 1.58 x 10^-17^; 20 μM: z-score = -4.00, p-value = 1 x 10^-12^). Treatment with 10 μM 680C91 reduced the abundance of nuclear SIRT6- and SIRT7-related targets GPAA1, MRPL16, MRPS31, MRPS33, and RPS3 (**Table S3**). At 20 μM 680C91, a change in SIRT7 was again identified at the pathway level (**Table S4**), although the directionality of the effect was ambiguous.

We were intrigued by the identification of SIRT7 specifically because of its multifaceted role in regulating chromatin condensation, DNA repair dynamics, tolerance of endoplasmic reticulum (ER) stress, and mitochondrial homeostasis (1–6). One function of SIRT7 is to promote tolerance of ER stress by suppressing Myc activity and silencing expression of ribosomal proteins (2). Consistent with loss of SIRT7 function, we identified Myc as one of the top upstream regulatory factors activated by inhibition of TDO (10 μM: z-score = 5.68, p-value = 2.64 x 10^-18^; 20 μM: z-score = 7.11, p-value = 1.61 x 10^-15^) and the abundance of ribosomal proteins was increased in cells treated with both concentrations of 680C91 (**Tables S3-4**). Accordingly, IPA identified increased activation of eukaryotic initiation factor 2 (eIF2) translational control in cells treated with the TDO inhibitor (10 μM: z-score = 5.24, p-value = 1 x 10^-67^; 20 μM: z-score = 2.56, p-value = 1 x 10^-17^). At the higher concentration of 680C91, an alteration in mammalian Target of Rapamycin (mTOR) was noted at the pathway level (z-score = 0.83, p-value = 2.5 x 10^-36^;), and inhibition of RICTOR, a component of mTOR complex 2 (mTORC2), was identified as either the top or one of the top alterations to upstream regulators at both concentrations of the TDO inhibitor (10 μM: z-score = -7.08, p-value = 8.4 x 10^-14^; 20 μM: z-score = -7.94, p-value = 6.5 x 10^-31^). Accordingly, we observed a decrease in phosphorylation of the mTOR2 target pS473 Akt when T98G cells were treated with 680C91 (**Fig. S6**).

Sirtuins depend on available stores of NAD^+^ to effectively catalyze deacetylation of a wide range of protein targets (7), and glioma cells use KYN-derived quinolinic acid (QA) to replenish NAD^+^ stores, which protects tumor cells from genotoxic agents (8). NAD^+^ levels also support maintenance of genomic integrity. The protection against DNA damage afforded by sustained NAD^+^ levels is related in part to adequate substrate availability for the DNA repair mediator poly[ADP-ribose]polymerase 1 (PARP-1) with simultaneous disruption of NAD^+^ biosynthesis and base excision repair (BER) sensitizing glioma cells to TMZ (9). To support the IPA of the proteomics results, we probed for total lysine acetylation via immunoblotting to assess global changes in sirtuin action in response to TDO inhibition (**Fig. S6A**). We also checked for total PARylation levels (**Fig. S6B**). Consistent with NAD^+^ depletion and diminished deacetylase activity, we observed a pronounced increase in total acetylated lysine, including acetylated histones, when GBM cells were treated with 680C91 (**Fig. S6A**). Treatment with 680C91 also produced a concomitant decrease in global PARylation (**Fig. S6B**), indirectly suggestive of diminished NAD^+^ stores in cells.

TDO inhibition resulted in a depletion of nuclear proteins directly involved in NER (nucleotide excision repair) and double-strand break repair (DSBR), including ATM, Mre11, Nbs1, SMARCAL1/2/4, WRN, and a number of chromatin remodeling enzymes. At the pathway level, loss of NER-related factors was scored as more significant, but inhibition of DSBR was also considered to be of importance based on IPA (**Tables S3-S4**). Overall, TDO inhibition in T98G cells seemed to promote a reduction in the nuclear abundance of factors associated with the DNA damage response.

We next analyzed proteome-level changes in T98G cells exposed to the DNA alkylating/crosslinking agent BCNU (**Table S5**). The concentration of BCNU we used for the proteomic analyses was 125 μM, which is below the EC_50_ value of ~200 μM that we measured for T98G cells but high enough to induce a response to DNA damage. Treating GBM-derived cells with BCNU led to activation of phosphatase and tensin homolog on chromosome 10 (PTEN), a negative regulator of PI3K/Akt/mTOR signaling (z-score = 2.11, p-value = 0.000093). These findings were further corroborated by decreased pS473 Akt in cells treated with BCNU (**Fig. S6C**). In addition to regulation of the PI3K/Akt/mTOR cascade, PTEN physically associates with centromeres to protect them from breakage and loss of PTEN leads to defects in HR through failed recruitment of Rad51 to sites of damage (10, 11). BCNU treatment resulted in a decrease in mTOR and eIF4/p70 S6K signaling (**Fig. 4C**), consistent with PTEN activation and indicative of an overall reduction in protein synthesis.

BCNU treatment also resulted in a predicted down-regulation of SIRT6 and SIRT7 signaling (**Table S5**), even below that observed for cells treated with 680C91 alone (**Table S8**). There was an overall increase in acetylated lysine by immunoblotting (**Fig. S6A**). However, the level of histone acetylation appeared to diminish in response to BCNU (**Fig. S6A**, see band near 17 kDa marker). BCNU-induced hypoacetylation of histone H3 has been reported previously for glioma-derived cells (12). The effect of BCNU on sirtuin signaling may be related to increased consumption of NAD^+^ by PARP-1. Indeed, we observed elevated PARylation levels accompanied BCNU treatment (**Fig. S6B**). NER and BRCA1-related DNA repair factors were also diminished by treatment with BCNU (**Table S5**), perhaps owing to the attenuation of protein synthesis. The abundance of nuclear localized DNA repair factors was lower in BCNU-treated cells than in cells treated with 20 μM 680C91 (**Table S8**, NER: z-score = 3.43, p-value = 2.5 x 10^-23^; BRCA1 DDR: z-score = 2.67, p-value = 1.58 x 10^-10^). Another difference between BCNU-treated cells and cells treated with 680C91 was that treatment with BCNU led to a slightly diminished Nrf2 antioxidant response (z-score = -0.66, p-value = 2 x 10^-15^) and a slight increase in SUMOylation (z-score = 0.96, p-value = 2.0 x 10^-13^).

Several interesting changes in the nuclear-enriched proteome were identified when we compared the results for cells treated with BCNU alone to those obtained with cells treated with the TDO inhibitor 680C91 (20 μM) prior to BCNU exposure (**Fig. 4D** and **E**, **Table S8**). At the pathway level, the most significant change identified was a decrease in tRNA charging (z-score = -3.4, p-value = 5 x 10^-18^). Based on quantitative proteomics, multiple tRNA synthetases, including Tyrosyl-tRNA synthetase (TyrRS), were depleted by co-treatment with 680C91 and BCNU relative to treatment with BCNU alone. This observation was interesting given that nuclear localized TyrRS was reported to upregulate expression of DNA repair factors, such as BRCA1 and RAD51, in response to oxidative stress through a direct interaction with the E2F1 transcription factor (13). Cells that cannot import TyrRS exhibit higher levels of γH2AX following treatment with H_2_O_2_ (13, 14).

Compared to treatment with BCNU alone, OXPHOS, Nrf2, acetyl-CoA biosynthesis, and eIF2 pathways were elevated by the pre-treatment with 680C91 prior to BCNU exposure (**Fig. 4E**), indicative of sustained energetic demands similar to what we observed for treatment with 680C91 alone. Combining TDO inhibition with BCNU resulted in diminished sirtuin signaling relative to BCNU alone (z-score = -2.46, p-value = 5 x 10^-11^). While total lysine acetylation for the combined treatment did not change much compared to BCNU alone, there was a slight increase in histone acetylation (**Fig. S5A**), which could signal diminished deacetylase action on chromatin in GBM cells with suppressed KP are exposed to BCNU.

Once again, SIRT7 was singled out as the major sirtuin family member regulating multiple proteins identified in our analysis (z-score = -2.02, p-value = 1.8 x 10^-5^). Nuclear abundance of SIRT7 was decreased by the combination treatment, as compared to BCNU treatment alone (**Fig. 4F**, log_2_FC = -0.32, p-value = 0.0024). As before, we observed activation of the sirtuin-regulated UPR mediator XBP1 (z-score = 5.48, p-value = 5.9 x 10^-9^). EGFR signaling was also elevated, as we observed with treatment with 680C91 alone (z-score = 2.33, p-value = 0.037), with EGFR abundance increasing slightly (log_2_FC = 0.44, p-value = 0.002). RICTOR-associated mTORC2 was predicted to be down-regulated in cells exposed to 680C91 and BCNU compared to BCNU alone (z-score = -2.64, p-value = 1.8 x 10^-5^). This prediction was further supported by lower pS473 Akt in T98G cells exposed to the combination treatment compared to BCNU alone (**Fig. S6C**), as well as a predicted activation of FOXO1 (z-score = 3.16, p-value = 8.0 x 10^-9^), which is normally suppressed by mTORC2.

Further interrogation of the proteomics data allowed us to identify additional changes in DSBR that extend beyond SIRT7-mediated effects. Some of these changes were consistent with the idea that loss of TDO activity results in a diminished capacity to repair BCNU-induced DNA damage. For example, there was a decrease in nuclear abundance of 53BP1 in cells treated with the combination of 680C91 and BCNU compared to treatment with BCNU alone (**Fig. 4F**). 53BP1 is a critical factor in repair of DSBs (15). Consistent with diminished 53BP1, there was an increase in BRCA1 and the associated E3 ubiquitin ligase UHRF1, which together promote K63-linked poly-ubiquitinylation of the 53BP1-binding partner Rif1 and suppress NHEJ (16). Furthermore, there was a broad reduction in proteins known to regulate repair of DSBs in a poly[ADP-ribose] (PAR)-dependent manner through interactions involving low complexity domains (LCDs). The PAR-dependent accumulation of LCD proteins induces liquid-liquid phase separation (i.e., biomolecular condensates) near sites of DNA damage (17). There was an uncanny similarity between the list of LCD-containing proteins depleted in 680C91/BNCU-treated cells and those previously implicated in PAR-mediated regulation of DNA repair through liquid-liquid demixing (17–20). This list included HNRNPD, HNRNPA1, HNRNPUL2, SAFB1, SAFB2, TAF15, RBM12B, RBM14, RBM15B, RBMX, and others (**Fig. 4F**). PAR-initiated liquid-liquid demixing nucleates self-assembling structures that may regulate DNA repair choice by filtering which factors gain access to sites of damage. In our experiments, the decreased nuclear abundance of these LCD-containing proteins may be related to limited PARP activity resulting from inadequate NAD^+^ stores, which could prevent effective liquid demixing and proper coordination of DNA repair.

The down-regulation of factors that control 53BP1 trafficking was also noted. Nucleoporin 153 (NUP153) was depleted, as was the nuclear structural protein NuMa 1 (**Fig. 4F**). NuMa1 was recently shown to control diffusion of 53BP1 (21). More specifically, NuMa1 was shown to reduce 53BP1 motility outside of DNA repair foci with no effect on total 53BP1 levels. Multiple lines of evidence support the notion that NUP153 is a key regulator of 53BP1 nuclear entry (22–25). The mechanism of NUP153-mediated import relies on the intermediate filament protein lamin A. Mislocalization of NUP153 in response to diminished lamin A (i.e., elevated levels of the lamin A precursor) leads to decreased localization of Ran, a GTPase responsible for nuclear import, and impeded nuclear entry of large protein cargo, such as 53BP1, but not smaller cargo like PCNA (24). The abundance of lamin A and Ran levels were found to be less in the cells treated with a combination of 680C91 and BCNU than in cells treated with BCNU alone (**Fig. 4F**). In line with this model, nuclear PCNA levels increased slightly when TDO inhibition was combined with BCNU (**Fig. 4F**). Analyzing a plot of the fold-change in protein abundance as a function of molecular weight produced a Pearson r value of -0.18 (p-value < 0.0001, **Fig. S7**), indicative of a modest negative effect on nuclear abundance for higher molecular weight proteins in cells treated with 680C91 and BCNU. It may be that down-regulation of KP signaling impairs nuclear entry for large cargo in response to BCNU treatment. This notion fits with a decrease in the nuclear abundance of 53BP1, a protein of >200 kDa, as well as decreased abundance for several other key repair factors (e.g., AATF, Mre11, ERCC4, FANCA, PolD1, Rad54B, RecQL, WRNIP). With that said, there were also increases in some high molecular weight repair factors, including FANCD2, FANCI, HLTF, and Rad18, which paints a more complicated picture of how DNA repair proteins are transported in GBM cells with an attenuated KP signaling cascade.

Another interesting difference between the BCNU and BCNU/680C91 treated samples was an overall increase in DNA replication-associated proteins when TDO activity was inhibited. In addition to PCNA, we observed increase in the relative abundance of RFC1-5, POLD1, PRIM1, PRIM2, and TOP2A (**Fig. 4F**). Slight increases in MCM2-7 were also apparent. There was a notable depletion of MCM3AP, which acetylates MCM3 and inhibits replication initiation (26). Mutations in MCM3AP have been shown to result in defective HR-mediated repair of DSBs (27), and failed activation of canonical NF- κB signaling caused by MCM3AP mutation may be responsible for the defect in HR repair. Such a scenario is consistent with the break repair defects we observed for TDO-deficient cells exposed to BCNU (**Fig. 2**).

There was >three-fold increase in cyclin A2 (CCNA2) in cells treated with 680C91 prior to BCNU exposure (**Fig. 4F**). A recent proteomics study identified cyclin A2 as one of the top PCNA-interactors following treatment with camptothecin (CPT) (28). The same study identified widely interspaced zinc finger (WIZ) as a PCNA-interacting partner. The authors speculated that WIZ might aid in the recruitment of the G9a-GLP methyltransferase. The action of the G9a-GLP methyltransferase facilitates recruitment of the BRCA1-associated E3 ligase UHRF1. UHRF1 is also an essential factor in the maintenance of DNA methylation (29). In accordance with these previous studies, we observed a small increase in nuclear WIZ and an approximate 3-fold increase in nuclear UHRF1 when we compared cells treated with BCNU alone to those treated with both 680C91 and BCNU (**Fig. 4F**).

We observed an increase in both CDK1 and CDK2 in the co-treated cells, again consistent with a relative acceleration in the replication program of TDO-deficient cells damaged with BCNU. Cyclin A2-CDK1 promotes origin firing, S-phase progression and mitotic entry (30). Cyclin A2 also helps coordinate mitotic entry through an interaction with CDK2 that promotes activation of the Anaphase Promoting Complex/Cyclosome (APC/C) (31). The overall increase in replication factors was coupled with important defects in DNA repair capacity noted above (e.g., loss of 53BP1, dysregulation of factors involved in PAR-initiated liquid-liquid demixing). These results may provide clues to understanding the elevated DNA damage and CIN observed in the cells co-treated with 680C91 and BCNU.

**TABLE S1. KEY RESOURCE AND REAGENT LIST**

| REAGENT or RESOURCE | DESIGNATION | SOURCE | IDENTIFIER | DILUTION |
| --- | --- | --- | --- | --- |
| **Primary Antibodies** | | | | |
| Rabbit anti-53BP1 | 53BP1 | Cell Signaling Technology | Cat# 4937 | 1:200 |
| Rabbit polyclonal anti-Akt | Akt | Cell Signaling  Technology | Cat# 9272 | 1:1000 |
| Rabbit polyclonal anti-phospho-Akt (Ser 473) | p-Akt | Cell Signaling  Technology | Cat# 4060 | 1:1000 |
| Rabbit polyclonal anti-ATM | ATM | Cell Signaling  Technology | D2E2; Cat# 2873 | 1:750 |
| Rabbit polyclonal anti-Akt | Akt | Cell Signaling  Technology | Cat# 9272 | 1:1000 |
| Rat anti-Bromodeoxyuridine | BrdU (CldU) | Novus Biologicals | Cat# NB500-169 | 1:250 |
| Mouse anti-Bromodeoxyuridine | BrdU (IdU) | BD Biosciences | (B44) Cat# 347580 | 1:250 |
| Rabbit polyclonal anti-phospho-Chk1 (Ser 345) | p-Chk1 | Cell Signaling  Technology | 133D3; Cat# 2348 | 1:1000 |
| Rabbit polyclonal anti-Chk2 | Chk2 | Abcam | Cat# Ab109413 | 1:1000 |
| Rabbit polyclonal anti-phospho-Chk2 (Thr 68) | p-Chk2 | Cell Signaling  Technology | C13C1; Cat# 2197 | 1:1000 |
| Rabbit polyclonal anti-GAPDH | GAPDH | Cell Signaling Technology | 14C10; Cat# 2118 | 1:10000 |
| Rabbit polyclonal anti-histone H3 | H3 | Cell Signaling  Technology | D1H2; Cat# 4499 | 1:2000 |
| Rabbit polyclonal anti-acetyl histone H3 (Lys 18) | Ac-H3 | Cell Signaling  Technology | Cat# 9675 | 1:750 |
| Rabbit polyclonal anti-gamma-H2AX (Ser 139) | γ-H2A.X | Abcam | Cat# ab81299 | 1:1000 (Western blotting & IF microscopy) |
| Mouse purified anti-H2A.X phospho (Ser139) | γ-H2A.X | BioLegend | Cat# 613401 | 1:200 (IF microscopy with 53BP1) |
| Rabbit anti-Acetylated-Lysine | panAc-Lys | Cell Signaling  Technology | Cat# 9441S | 1:1000 |
| Mouse monoclonal anti-poly (ADP-ribose) polymer | PAR | Abcam | 10H; Cat# ab14459 | 1:1000 |
| Mouse monoclonal anti-PARP-1 | PARP1 | Santa Cruz Biotech | F2; Cat# sc-8007 | 1:1000 |
| Rabbit polyclonal anti-RPA2 (Ser 33) | RPA2(s33) | Novus Biologicals | Cat# NB100-544 | 1:250 |
| Mouse monoclonal anti-SIRT-7 | SIRT7 | Santa Cruz Biotech | C3; Cat# sc-365344 | 1:1000 |
| Mouse polyclonal anti-TDO2 | TDO2 | Abnova | Cat# H00006999-BO1P | 1:1000 |
| Mouse monoclonal anti-α-Tubulin | α-Tubulin | MiliporeSigma | Cat# T9026 | 1:500 |
| **Secondary Antibodies** | | | | |
| Goat anti-Rat IgG,  Alexa Fluor 594 | AF594 | Invitrogen | Cat# A-11007 | 1:100 |
| Goat anti-Rabbit IgG,  Alexa Fluor 647 | AF647 | ThermoFisher Scientific | Cat# A-21244 | 1:100 |
| Goat anti-Mouse IgG, DyLight 488 | DyLight 488 | ThermoFisher Scientific | Cat# 35502 | 1:100 |
| Goat anti-Mouse IgG,  DyLight 650 | DyLight 650 | ThermoFisher Scientific | Cat# 84545 | 1:50 |
| Goat polyclonal anti-Mouse HRP conjugated | Anti-mouse | ThermoFisher Scientific | Cat# 32430 | 1:2000 – 1:5000 |
| Goat polyclonal anti-Rabbit HRP conjugated | Anti-rabbit | ThermoFisher Scientific | Cat# 32460 | 1:2000 – 1:5000 |
| **siRNA** | | | | |
| On-TARGETplus Non-targeting control pool | Horizon Discovery | | Cat# D-001810-10-05 | |
| **Target Sequences** | | | | |
| 5ʹ-UGGUUUACAUGUCGACUAA-3ʹ, 5ʹ-UGGUUUACAUGUUGUGUGA-3ʹ, 5ʹ-UGGUUUACAUGUUUUCUGA-3ʹ, 5ʹ-UGGUUUACAUGUUUUCCUA-3ʹ | | | | |
| On-TARGETplus human TDO2 siRNA SMARTpool | Horizon Discovery | | Cat# L-008506-01-0005 | |
| **Target Sequences** | | | | |
| 5ʹ-UCAUAAGGAUUCAGGCUAA-3ʹ, 5ʹ-AGUGAUAGGUACAAGGUAU-3ʹ, 5ʹ-GGAUUUAACUUCUGGGGAA-3ʹ, 5ʹ-GCGAAGAAGACAAAUCACA-3ʹ | | | | |


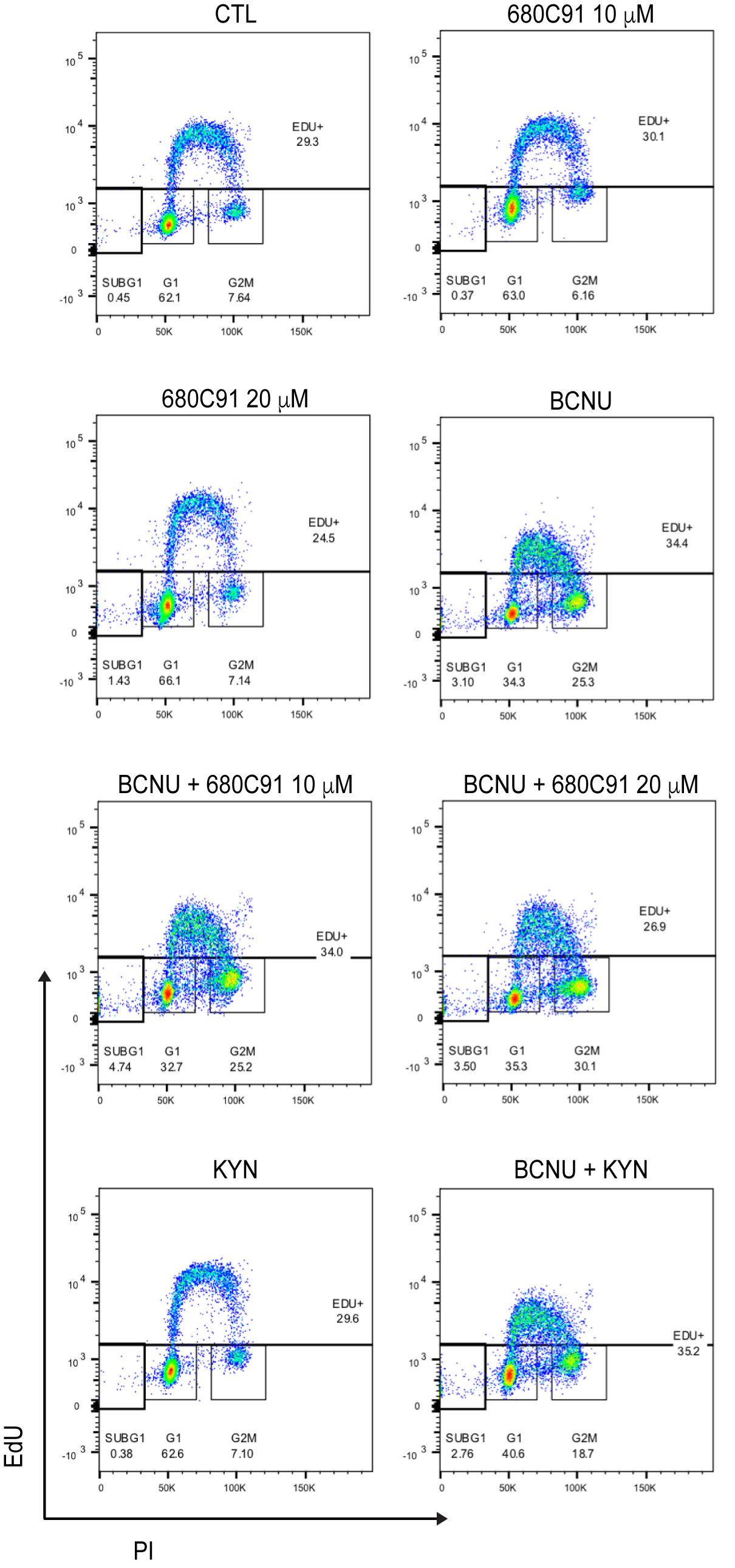


**FIGURE S1. Representative flow-cytometry results.** Scatterplots are representative for treatments shown in Figure 3B, where the Y-axis depicts EdU positive cells and the X-axis depicts PI staining.

**
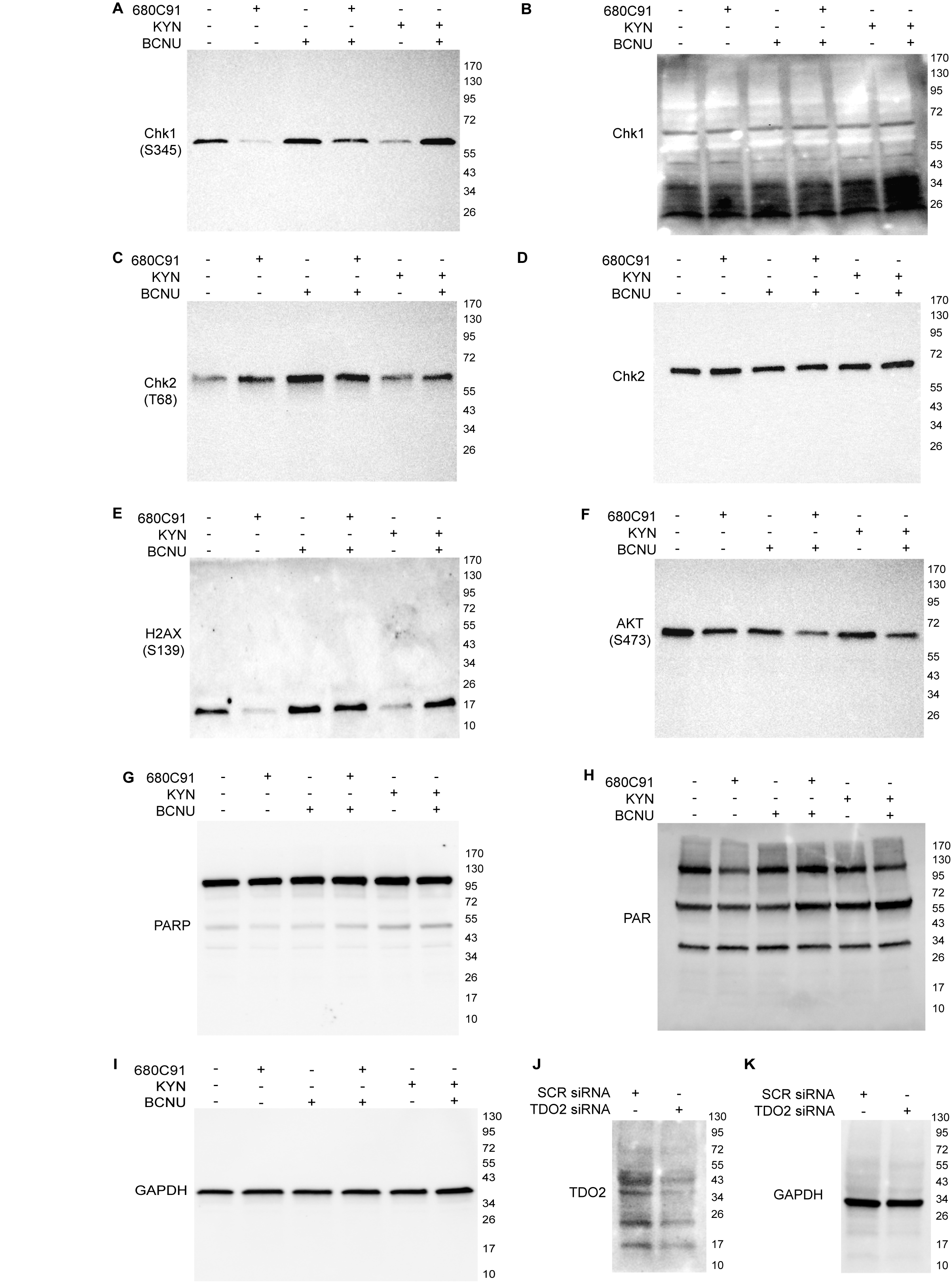
**

**FIGURE S2. Uncropped immunoblots for whole cell lysate (WCL) samples.** Images of full blots of the samples shown as cropped images in Figure 1H and Figure S3. The images shown are one of two replicates. The primary antibody used for probing each blot is indicated to the left of each panel, and the positions of bands for the reference molecular weight ladder are indicated towards the right of each blot.

**
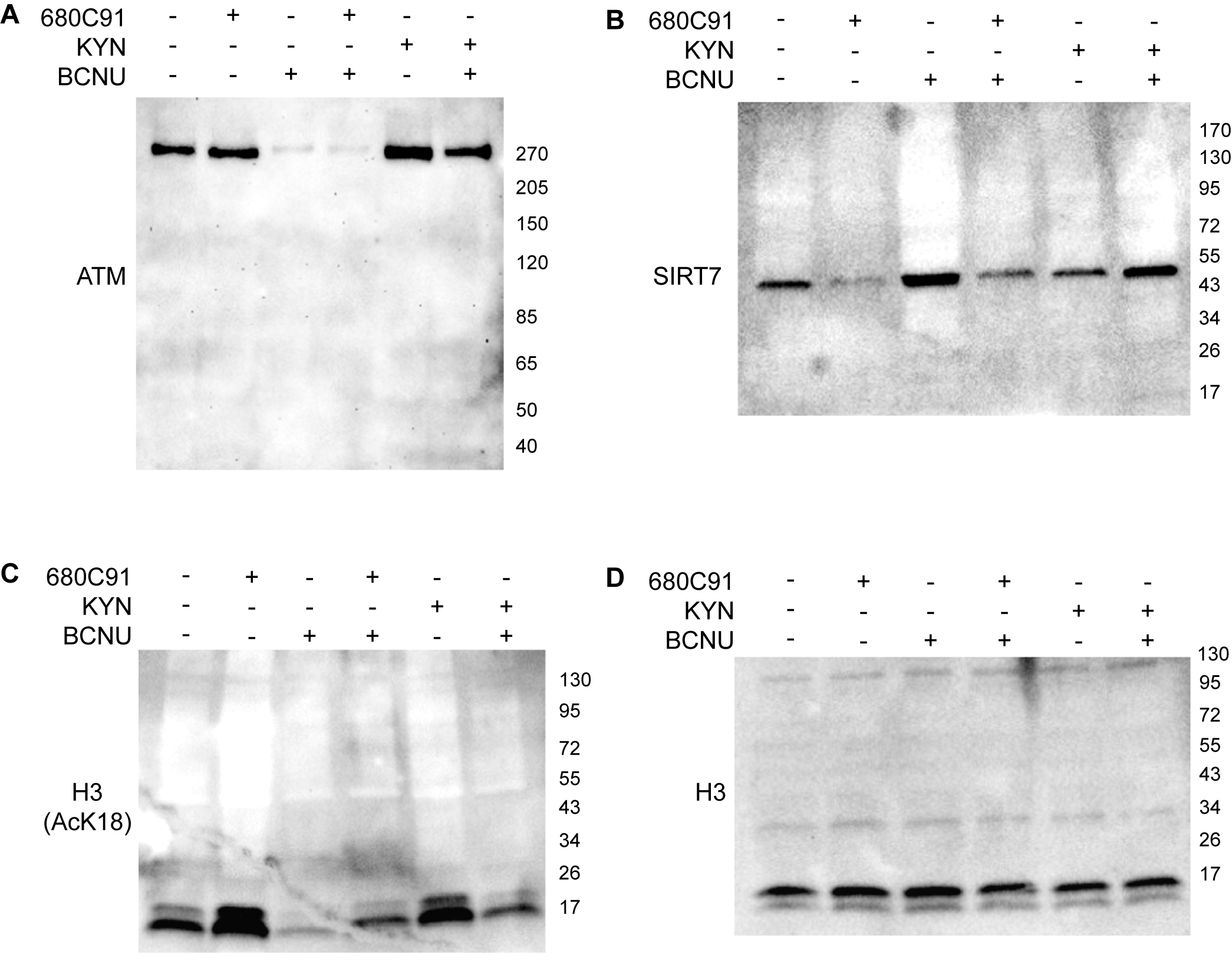
**

**FIGURE S3. Uncropped immunoblots for chromatin-bound (CB) samples.** Images of full blots of the samples shown as cropped images in Figure 6 of the main text. The primary antibody used for probing each blot is indicated above each panel, and the positions of bands for the reference molecular weight ladder are indicated towards the left of each blot.

**
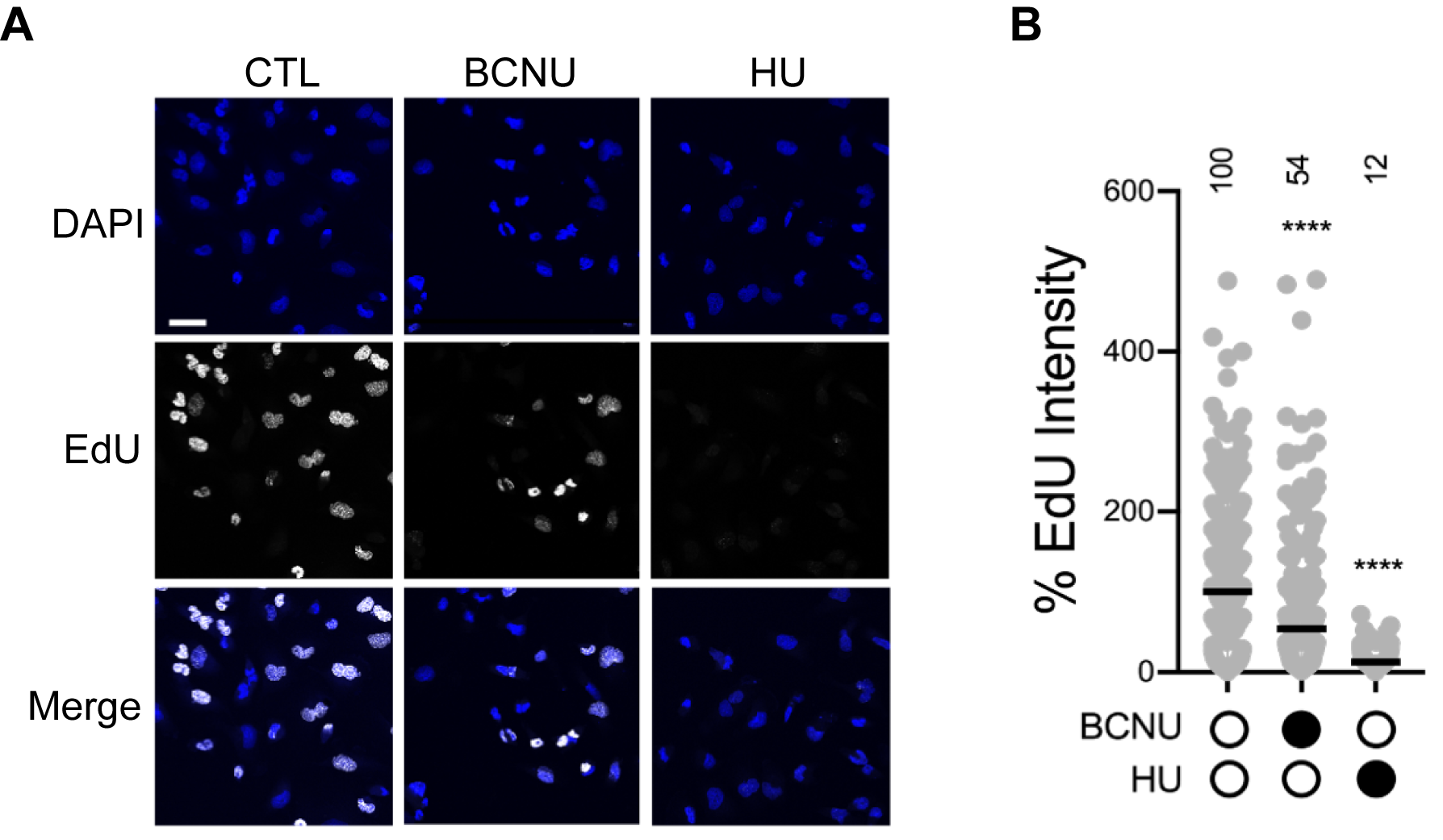
**

**FIGURE S4. T98G EdU incorporation immunofluorescence experiment.**

*A,* Representative immunofluorescent images of T98G cells co-treated with 10 μM EdU ± BCNU (125 μM) or HU (2 mM) for 1 h. *B,* Graph of percentage of EdU intensity relative to control treated cells. Where mean percent fluorescent intensity is depicted above each condition. Experiment was conducted in biological duplicate with at least 300 cells scored per experimental condition. Statistical analysis was calculated using one-way ANOVA with Tukey post-test, where * is P≤ 0.05, ** is P≤ 0.01, *** is P ≤ 0.001 and **** is P ≤ 0.0001.


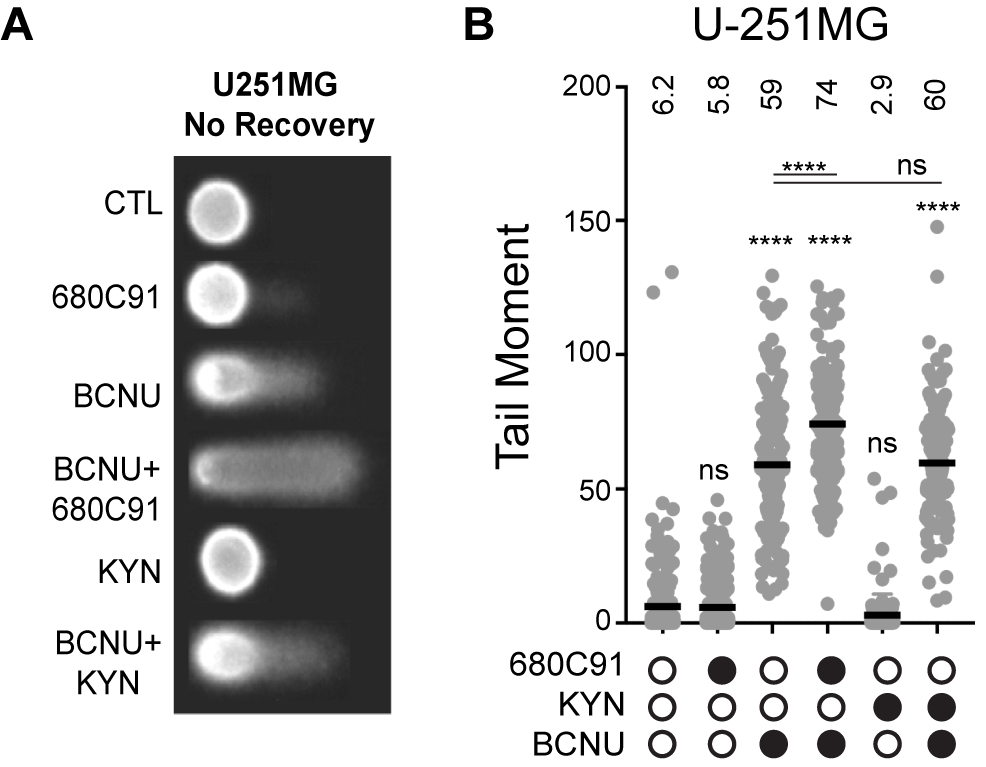


**FIGURE S5. U-251MG comet assay results.** *A,* Representative comet assay images for U-251MG cells treated +/- 680C91 (20 μM), KYN (60 μM), BCNU (125 μM) or a combination of these compounds. *B,* Tail moment of U-251MG cells treated as described in *A*. Mean tail moment is depicted above each experimental condition, and at least 130 cells were scored. Statistical analysis was calculated using one-way ANOVA with Tukey post-test, where * is P≤ 0.05, ** is P≤ 0.01, *** is P ≤ 0.001 and **** is P ≤ 0.0001.

**
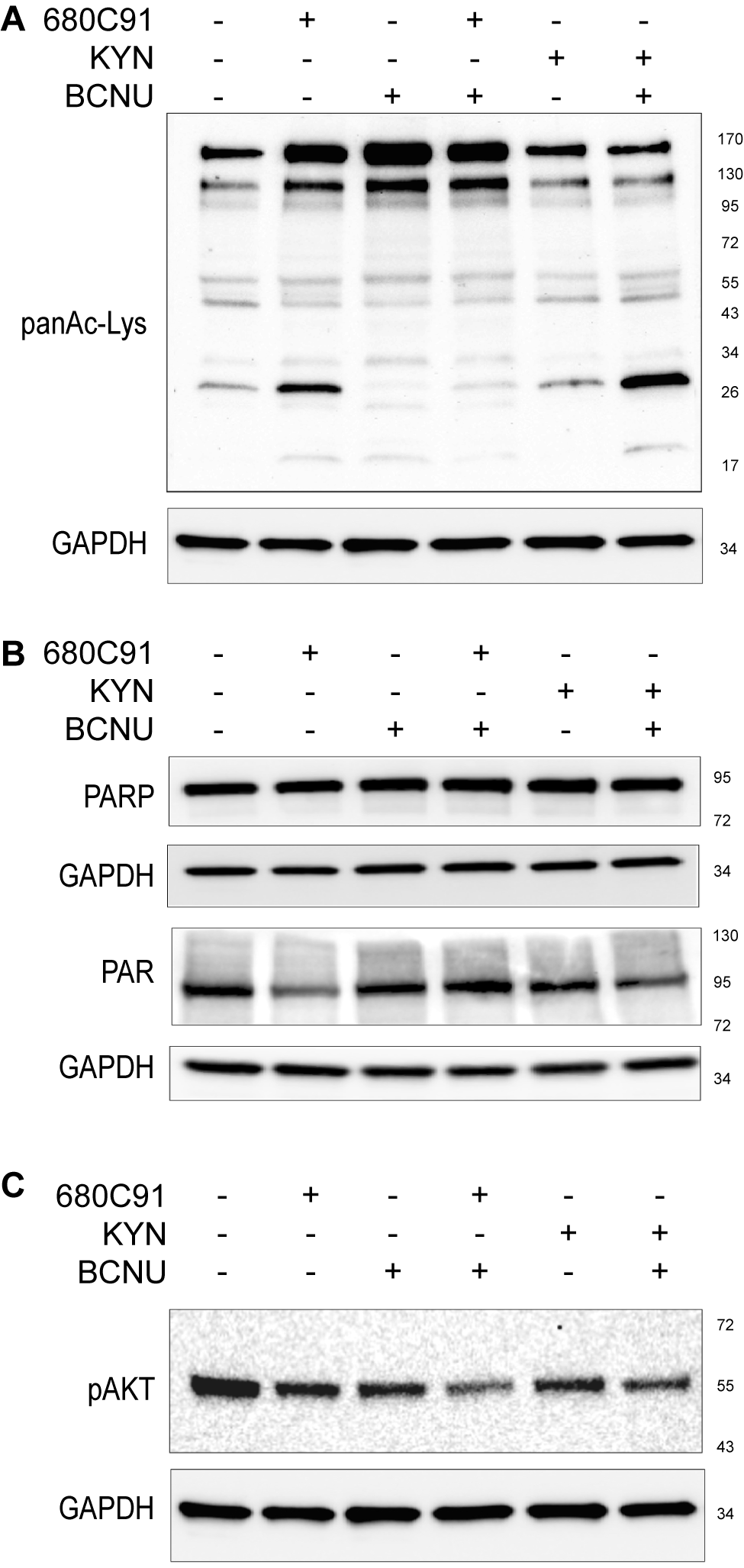
**

**FIGURE S6. Immunoblotting revealed that TDO inhibition resulted in decreased deacetylase activity, PAR-ylation, and phospho-AKT levels.**

A, Immunoblot of panAcetyl-Lysine expression.

B, Immunoblot of total PARP and PAR-ylation levels.

C, Immunoblot of phosphorylated AKT (S473).


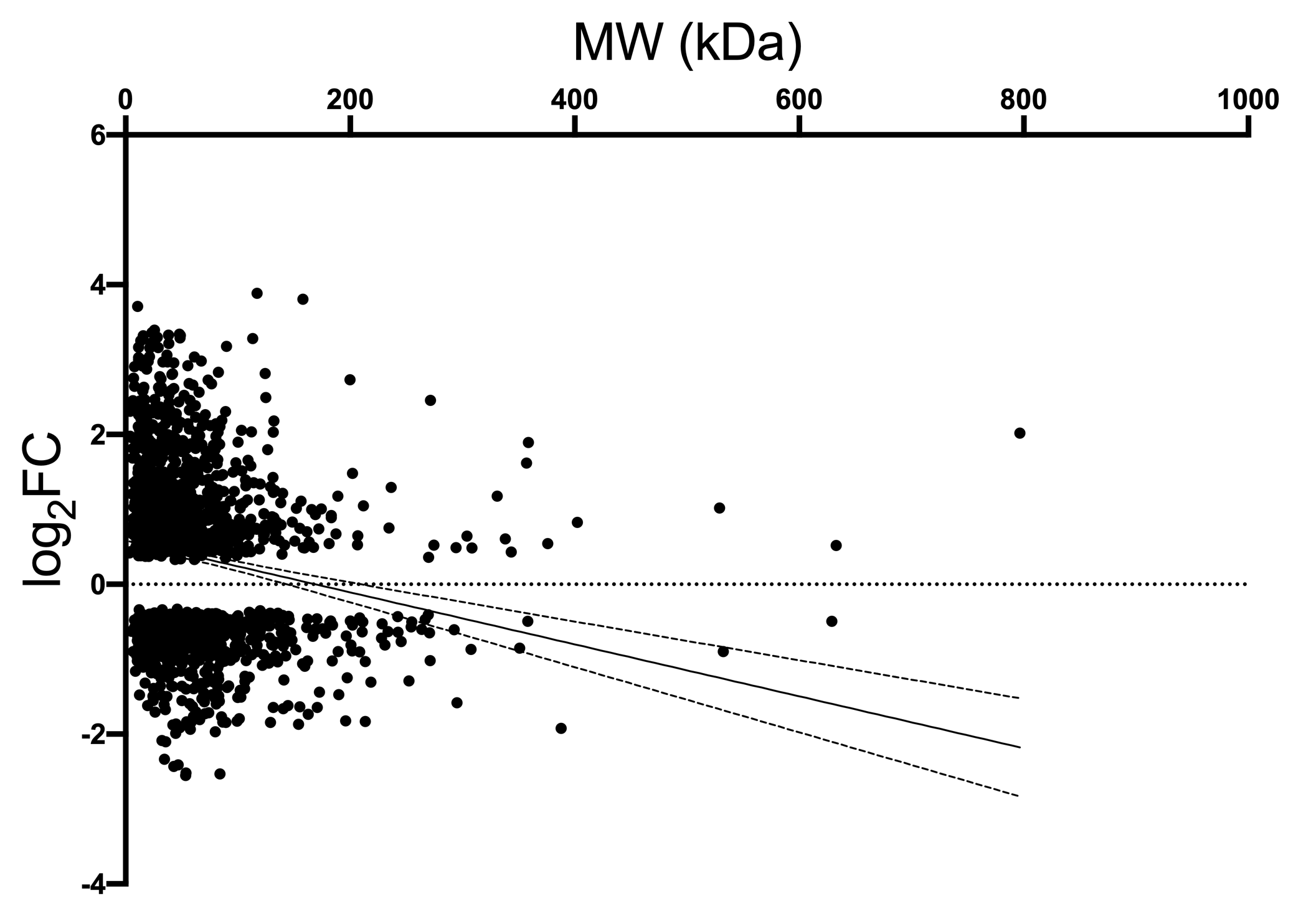


**FIGURE S7. Quantification of changes in protein abundance as a function of molecular weight.** The log_2_ fold-change (FC) for all proteins with an FDR-adjusted p-value of <0.05 are shown plotted as a function of molecular weight in kDa. Simple linear regression was used to provide an initial directionality to the resulting plot. The resulting line is shown along with the 95% confidence intervals (dashed lines). To evaluate correlation between the direction of change and protein molecular weight, a Pearson correlation value was calculated using GraphPad Prism – resulting in a Pearson r value = -0.1852 (95% confidence interval of -0.2316 to -0.1380, R^2^ = 0.034) and a two-tailed P value of <0.0001.

REFERENCES

1. Vazquez,B.N., Thackray,J.K. and Serrano,L. (2017) Sirtuins and DNA damage repair: SIRT7 comes to play. *Nucl.*, **8**, 107–115.

2. Shin,J., He,M., Liu,Y., Paredes,S., Villanova,L., Brown,K., Qiu,X., Nabavi,N., Mohrin,M., Wojnoonski,K., *et al.* (2013) SIRT7 represses Myc activity to suppress ER stress and prevent fatty liver disease. *Cell Rep.*, **5**, 654–665.

3. Tang,M., Li,Z., Zhang,C., Lu,X., Tu,B., Cao,Z., Li,Y., Chen,Y., Jiang,L., Wang,H., *et al.* (2019) SIRT7-mediated ATM deacetylation is essential for its deactivation and DNA damage repair. *Sci. Adv.*, **5**, eaav1118.

4. Song,C., Hotz-Wagenblatt,A., Voit,R. and Grummt,I. (2017) SIRT7 and the DEAD-box helicase DDX21 cooperate to resolve genomic R loops and safeguard genome stability. *Genes Dev.*, **31**, 1370–1381.

5. Li,H., Tian,Z., Qu,Y., Yang,Q., Guan,H., Shi,B., Ji,M. and Hou,P. (2019) SIRT7 promotes thyroid tumorigenesis through phosphorylation and activation of Akt and p70S6K1 via DBC1/SIRT1 axis. *Oncogene*, **38**, 345–359.

6. Yu,J., Qin,B., Wu,F., Qin,S., Nowsheen,S., Shan,S., Zayas,J., Pei,H., Lou,Z. and Wang,L. (2017) Regulation of Serine-Threonine Kinase Akt Activation by NAD+-Dependent Deacetylase SIRT7. *Cell Rep.*, **18**, 1229–1240.

7. Dai,H., Sinclair,D.A., Ellis,J.L. and Steegborn,C. (2018) Sirtuin activators and inhibitors: Promises, achievements, and challenges. *Pharmacol. Ther.*, **188**, 140–154.

8. Sahm,F., Oezen,I., Opitz,C.A., Radlwimmer,B., von Deimling,A., Ahrendt,T., Adams,S., Bode,H.B., Guillemin,G.J., Wick,W., *et al.* (2013) The endogenous tryptophan metabolite and NAD+ precursor quinolinic acid confers resistance of gliomas to oxidative stress. *Cancer Res.*, **73**, 3225–3234.

9. Goellner,E.M., Grimme,B., Brown,A.R., Lin,Y.-C., Wang,X.-H., Sugrue,K.F., Mitchell,L., Trivedi,R.N., Tang,J. and Sobol,R.W. (2011) Overcoming temozolomide resistance in glioblastoma via dual inhibition of NAD+ biosynthesis and base excision repair. *Cancer Res.*, **71**, 2308–2317.

10. Shen,W.H., Balajee,A.S., Wang,J., Wu,H., Eng,C., Pandolfi,P.P. and Yin,Y. (2007) Essential role for nuclear PTEN in maintaining chromosomal integrity. *Cell*, **128**, 157–170.

11. Bassi,C., Ho,J., Srikumar,T., Dowling,R.J.O., Gorrini,C., Miller,S.J., Mak,T.W., Neel,B.G., Raught,B. and Stambolic,V. (2013) Nuclear PTEN controls DNA repair and sensitivity to genotoxic stress. *Science*, **341**, 395–399.

12. Papait,R., Magrassi,L., Rigamonti,D. and Cattaneo,E. (2009) Temozolomide and carmustine cause large-scale heterochromatin reorganization in glioma cells. *Biochem. Biophys. Res. Commun.*, **379**, 434–439.

13. Wei,N., Shi,Y., Truong,L.N., Fisch,K.M., Xu,T., Gardiner,E., Fu,G., Hsu,Y.-S.O., Kishi,S., Su,A.I., *et al.* (2014) Oxidative stress diverts tRNA synthetase to nucleus for protection against DNA damage. *Mol. Cell*, **56**, 323–332.

14. Cao,X., Li,C., Xiao,S., Tang,Y., Huang,J., Zhao,S., Li,X., Li,J., Zhang,R. and Yu,W. (2017) Acetylation promotes TyrRS nuclear translocation to prevent oxidative damage. *Proc. Natl. Acad. Sci. U. S. A.*, **114**, 687–692.

15. Panier,S. and Boulton,S.J. (2014) Double-strand break repair: 53BP1 comes into focus. *Nat. Rev. Mol. Cell Biol.*, **15**, 7–18.

16. Zhang,H., Liu,H., Chen,Y., Yang,X., Wang,P., Liu,T., Deng,M., Qin,B., Correia,C., Lee,S., *et al.* (2016) A cell cycle-dependent BRCA1-UHRF1 cascade regulates DNA double-strand break repair pathway choice. *Nat. Commun.*, **7**, 10201.

17. Altmeyer,M., Neelsen,K.J., Teloni,F., Pozdnyakova,I., Pellegrino,S., Grøfte,M., Rask,M.-B.D., Streicher,W., Jungmichel,S., Nielsen,M.L., *et al.* (2015) Liquid demixing of intrinsically disordered proteins is seeded by poly(ADP-ribose). *Nat. Commun.*, **6**, 8088.

18. Simon,N.E., Yuan,M. and Kai,M. (2017) RNA-binding protein RBM14 regulates dissociation and association of non-homologous end joining proteins. *Cell Cycle*, **16**, 1175–1180.

19. Polo,S.E., Blackford,A.N., Chapman,J.R., Baskcomb,L., Gravel,S., Rusch,A., Thomas,A., Blundred,R., Smith,P., Kzhyshkowska,J., *et al.* (2012) Regulation of DNA-end resection by hnRNPU-like proteins promotes DNA double-strand break signaling and repair. *Mol. Cell*, **45**, 505–516.

20. Yuan,M., Eberhart,C.G. and Kai,M. (2014) RNA binding protein RBM14 promotes radio-resistance in glioblastoma by regulating DNA repair and cell differentiation. *Oncotarget*, **5**, 2820–2826.

21. Moreno,N.S., Liu,J., Haas,K.M., Parker,L.L., Chakraborty,C., Kron,S.J., Hodges,K., Miller,L.D., Langefeld,C., Robinson,P.J., *et al.* (2019) The nuclear structural protein NuMA is a negative regulator of 53BP1 in DNA double-strand break repair. *Nucleic Acids Res.*, **47**, 10475.

22. Mackay,D.R., Howa,A.C., Werner,T.L. and Ullman,K.S. (2017) Nup153 and Nup50 promote recruitment of 53BP1 to DNA repair foci by antagonizing BRCA1-dependent events. *J. Cell Sci.*, **130**, 3347–3359.

23. Duheron,V., Nilles,N., Pecenko,S., Martinelli,V. and Fahrenkrog,B. (2017) Localisation of Nup153 and SENP1 to nuclear pore complexes is required for 53BP1-mediated DNA double-strand break repair. *J. Cell Sci.*, **130**, 2306–2316.

24. Cobb,A.M., Larrieu,D., Warren,D.T., Liu,Y., Srivastava,S., Smith,A.J.O., Bowater,R.P., Jackson,S.P. and Shanahan,C.M. (2016) Prelamin A impairs 53BP1 nuclear entry by mislocalizing NUP153 and disrupting the Ran gradient. *Aging Cell*, **15**, 1039–1050.

25. Moudry,P., Lukas,C., Macurek,L., Neumann,B., Heriche,J.-K., Pepperkok,R., Ellenberg,J., Hodny,Z., Lukas,J. and Bartek,J. (2012) Nucleoporin NUP153 guards genome integrity by promoting nuclear import of 53BP1. *Cell Death Differ.*, **19**, 798–807.

26. Takei,Y., Assenberg,M., Tsujimoto,G. and Laskey,R. (2002) The MCM3 acetylase MCM3AP inhibits initiation, but not elongation, of DNA replication via interaction with MCM3. *J. Biol. Chem.*, **277**, 43121–43125.

27. Gatz,S.A., Salles,D., Jacobsen,E.-M., Dörk,T., Rausch,T., Aydin,S., Surowy,H., Volcic,M., Vogel,W., Debatin,K.-M., *et al.* (2016) MCM3AP and POMP Mutations Cause a DNA-Repair and DNA-Damage-Signaling Defect in an Immunodeficient Child. *Hum. Mutat.*, **37**, 257–268.

28. Srivastava,M., Chen,Z., Zhang,H., Tang,M., Wang,C., Jung,S.Y. and Chen,J. (2018) Replisome Dynamics and Their Functional Relevance upon DNA Damage through the PCNA Interactome. *Cell Rep.*, **25**, 3869-3883.e4.

29. Ferry,L., Fournier,A., Tsusaka,T., Adelmant,G., Shimazu,T., Matano,S., Kirsh,O., Amouroux,R., Dohmae,N., Suzuki,T., *et al.* (2017) Methylation of DNA Ligase 1 by G9a/GLP Recruits UHRF1 to Replicating DNA and Regulates DNA Methylation. *Mol. Cell*, **67**, 550-565.e5.

30. Katsuno,Y., Suzuki,A., Sugimura,K., Okumura,K., Zineldeen,D.H., Shimada,M., Niida,H., Mizuno,T., Hanaoka,F. and Nakanishi,M. (2009) Cyclin A-Cdk1 regulates the origin firing program in mammalian cells. *Proc. Natl. Acad. Sci. U. S. A.*, **106**, 3184–3189.

31. Hein,J.B. and Nilsson,J. (2016) Interphase APC/C-Cdc20 inhibition by cyclin A2-Cdk2 ensures efficient mitotic entry. *Nat. Commun.*, **7**, 10975.
